# Clues to transcription/replication collision-induced DNA damage: it was RNAP, in the chromosome, with the fork

**DOI:** 10.1002/1873-3468.15063

**Published:** 2024-11-24

**Authors:** Matthew B. Cooke, Christophe Herman, Priya Sivaramakrishnan

**Affiliations:** 1 Department of Molecular and Human Genetics, Baylor College of Medicine, Houston, TX, USA; 2 Department of Molecular Virology and Microbiology, Baylor College of Medicine, Houston, TX, USA; 3 Dan L. Duncan Cancer Center, Baylor College of Medicine, Houston, TX, USA; 4 Center for Computational and Genomic Medicine, Children’s Hospital of Philadelphia, PA, USA

**Keywords:** DNA repair, DNA replication, mutagenesis, replisome, RNA transcription, RNAP backtracking, transcription–replication collision

## Abstract

DNA replication and RNA transcription processes compete for the same DNA template and, thus, frequently collide. These transcription–replication collisions are thought to lead to genomic instability, which places a selective pressure on organisms to avoid them. Here, we review the predisposing causes, molecular mechanisms, and downstream consequences of transcription–replication collisions (TRCs) with a strong emphasis on prokaryotic model systems, before contrasting prokaryotic findings with cases in eukaryotic systems. Current research points to genomic structure as the primary determinant of steady-state TRC levels and RNA polymerase regulation as the primary inducer of excess TRCs. We review the proposed mechanisms of TRC-induced DNA damage, attempting to clarify their mechanistic requirements. Finally, we discuss what drives genomes to select against TRCs.

Cells must replicate their DNA accurately, to ensure proper information transfer across generations, as well as quickly, to complete replication during a defined period of the cell division cycle. To do this, cells depend on a complex and processive replication apparatus called the replisome. This replisome is a large, multiprotein complex that translocates across the genome, making a precise copy as it goes.

Disruptions to replisome functions are known to result in mutagenesis and compromise cellular fitness [1]. The major source of DNA replisome disruption is DNA-bound proteins [2,3], the most abundant of which is RNA polymerase (RNAP) [4]. As the replisome and RNAP are both large, processive complexes that share the same DNA template, they functionally compete for the same underlying DNA. This competition frequently results in collisions between the complexes, termed transcription–replication collisions or TRCs. We now appreciate TRCs as the primary impediment to replisome activity in the genome [2,5]. The central role of TRCs in understanding replisome behavior, DNA replication, and resulting genomic changes has associated TRCs with genome evolution [6] and global gene regulation [5].

It appears that much of the cellular effects of TRCs are explained by a small subset of collisions—with thousands of collisions only culminating in a handful of toxic TRCs per cell division cycle. As such, the study of TRCs has focused on diagnosing what predispositions and molecular mechanisms lead to a particular TRC being benign or toxic.

In this review, we summarize the current understanding of these predispositions and molecular mechanisms that lead to toxic TRCs. This review relies heavily on the study of *Escherichia coli* genome structure as well as replisome and RNAP behavior, as this model system composes the bulk of TRC literature—though studies performed in organisms such as *Bacillus subtilis*, *Caulobacter crescentus*, and eukaryotic models are cited, when appropriate. First, we introduce the three major players of the TRC interaction: the underlying genome structure, the replisome itself, and the various RNAP complex configurations (Fig. 1A). Second, we discuss what predisposing factors or stressors lead TRCs to happen in the first place and, when these stressors are intensified, cause TRCs to become more common (Fig. 1B). Third, we consider the various models for TRC-induced DNA damage (Fig. 1C). Finally, we discuss how TRCs alter cell fitness and their ultimate effects on genome evolution (Fig. 1D).

## The players

### The underlying genome structure

**Fig. 2.**
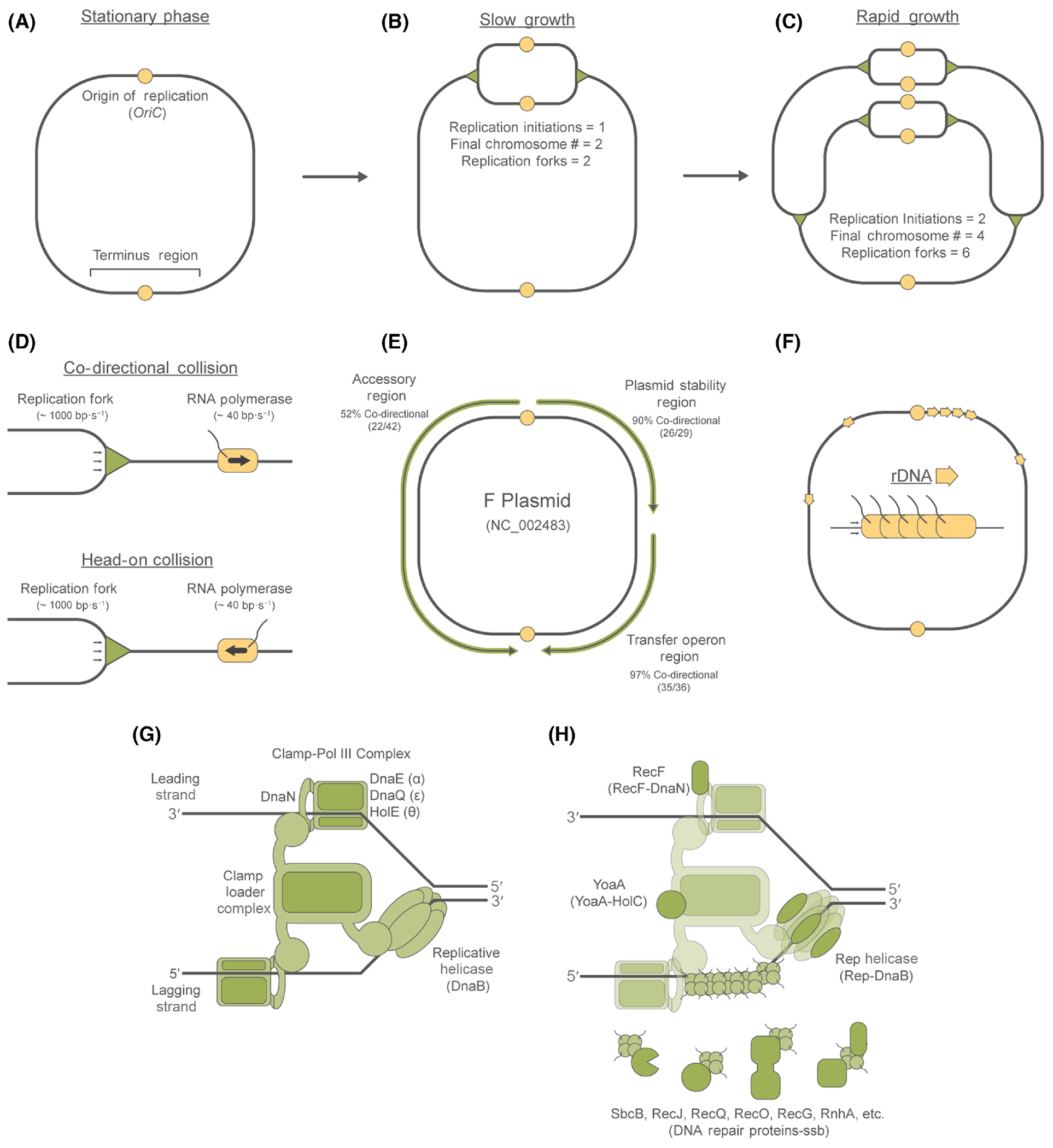
Prokaryotic genome structure potentiates replication–transcription interactions and subsequent evolution. (A) Prokaryotic genomes are circular, with a single replication origin (*oriC*) and a replication terminus region. (B) Initiation of prokaryotic DNA replication occurs from *oriC* bidirectionally. (C) During rapid growth, multiple subsequent rounds of DNA replication are initiated during the same cell division cycle. (D) DNA replication and RNA transcription can collide in two orientations, known as codirectional or head-on. Due to the rapid speed of prokaryotic replication forks, replisome–RNAP interactions are expected regardless of collision orientation. (E) Prokaryotic sequence elements such as plasmids and genomes strongly select for codirectionality between replication and transcription, suggesting a less deleterious collision outcome. (F) The most highly transcribed loci, rDNAs are under strong selection to orient codirectionally to replication. (G) The core DNA replication fork can be simplified into three parts: the replicative helicase, clamp loader complex, and the clamp-Pol III complex. (H) The core fork components recruit secondary factors that assist in avoiding, processing, and repairing transcription–replication collisions.

Prokaryotic genomes are mostly circular, haploid, and monochromosomal (Fig. 2A). Each chromosome contains a single origin of replication, *oriC*, which nucleates bidirectional sets of DNA replication forks (Fig. 2B). Due to the circular nature of the chromosome, each fork must replicate half of the chromosome and will reconvene at the replication terminus region to complete a single round of DNA replication. As one round of chromosomal DNA replication can take longer than a single cell division cycle, depending on the nutrient conditions [7] (*E. coli* replication cycle = 40 min; *E. coli* division cycle = 20 min in rich media), bacteria will initiate additional rounds of DNA replication prior to the initial replication cycle finishing, a process called multi-forked replication [8] (Fig. 2C). This differential between the rate of bacterial cell division and rate of DNA replication places a selective pressure on bacteria to evolve processive, fast replication forks. As such, the bacterial replication fork synthesizes DNA considerably faster than their eukaryotic counterparts (1000 bp·^−1^ vs. 50 bp·^−1^ on average) [9].

The structure and orientation of bacterial genomes has also evolved to streamline DNA replication [10]. There are two possible orientations of a transcription unit, relative to DNA replication: codirectional or head-on (Fig. 2D). Early observations found that many highly transcribed loci were oriented such that RNA transcription and DNA replication proceeded in the same direction, codirectionally, rather than head-on [11]. The subsequent age of genomics revealed that most bacterial sequences and sequence elements, including bacterial genomes, phage genomes, and plasmids, are heavily biased toward orienting genes codirectionally with DNA replication [12,13] (Fig. 2E). The most highly transcribed loci, rDNA loci, are universally oriented codirectionally with DNA replication and frequently cluster close to the origin of replication [14] (Fig. 2F). Subsequent analysis found positive correlations between gene transcription levels, expression levels [5], or essentiality [15] and the likelihood of gene codirectionality. For example, in the *E. coli* F plasmid (Refseq NC_002483), the conserved regions responsible for plasmid stability and mobility are 90% and 97% codirectional with DNA replication, compared to 52% for its nonconserved region (Fig. 2E). Wider observations across the bacterial tree of life have revealed that genomic bias toward gene codirectionality can be directly correlated with the evolution of distinct replicative DNA polymerases [16]. These observations suggest that genome structure evolves in response to interactions between DNA replication and RNA transcription.

### The replisome

When referring to the DNA replisome, we are considering two entities. The first is the core DNA replication complex in the context of DNA replication elongation (i.e., we will not discuss the initiation complex) (Fig. 2G). The second entity is the web of replisome-associated proteins that assist in the replication process (Fig. 2H). More thorough reviews of replisome composition [17] and replisome protein recruitment [18] are available. For the purposes of this review, we will introduce the relevant protein components and their functions.

The core DNA replication complex is composed of three interacting parts: the replicative helicase complex, the clamp loader complex, and the clamp-polymerase complex [17]. The replicative helicase complex is the stable core of the DNA replication complex [19,20]. It is composed of a homo-hexameric DnaB ssDNA helicase loaded onto the lagging strand of the replication fork. This helicase stays associated with its bound DNA for longer than any other fork component (tens of minutes per binding event) [20,21], directly interacts with the other two main fork components [22–24] (the clamp loader [22] and clamp-polymerase complex [23,24]), and its loading is sufficient to nucleate an entire DNA replisome during DNA replication initiation [25] and DNA replication restart [26]. Here, we will define replication fork ‘collapse’ as any event that results in dissociation of the DnaB helicase, thus exposing the replication fork junction and necessitating defined fork restart processes. This contrasts with the term, replication fork ‘stalling’, which refers to events that halt the forward progression of the DnaB helicase.

The clamp loader complex is a structural subunit of the core DNA replication complex. The clamp loader functions to load DnaN sliding clamps onto both the leading and lagging replication strands as well as encourage association of the DNA polymerase itself with the sliding clamp [23,24]. Clamp loader complex function ensures the proper nucleation of a core DNA replication complex and loading of replicative DNA polymerases onto the lagging strand during processive DNA replication.

The clamp–polymerase complex is responsible for catalytic addition of new dNTPs onto the nascent replication fork. It is composed of the DnaN β sliding clamp [27] as well as the Pol III, α (DnaE), ε (DnaQ), and θ (HolE) complex [28]. The sliding clamp’s primary function appears to allosterically regulate the elongation activity of the Pol III complex [28], though it has notable moonlighting functions related to DNA replication initiation [29] and alternative DNA polymerase recruitment [30]. Pol III α is the DNA catalytic subunit which stably associates with the ε proofreading subunit through an interaction stabilized by θ [17]. Pol III undergoes high levels of turnover at the replication fork [20,31] and its frequent dissociation does not appear to inhibit the continuous forward progression of the DNA replication fork [20,32,33]. Conversely, the sliding clamp undergoes less frequent turnover than other replication complex components, except for the replicative helicase [20].

Besides the core DNA replication complex, the second entity is composed of several replisome-associated proteins that are primarily recruited to the fork through interactions with DNA single-strand binding protein [18], Ssb, or direct interactions with core replication complex components [34]. Ssb associates with the DNA replication fork by binding lagging strand ssDNA prior to Okazaki fragment synthesis. Ssb is a small (178AA) tetramer with a high rate of 3D diffusion (unbound) [35] and 2D diffusion (along bound ssDNA) [36]. This property allows Ssb to function as a shuttle for DNA processing protein recruitment. Ssb direct interactions include core replication complex components and DNA repair enzymes such as SbcB, RecJ, RecQ, RecO, RecG, and RnhA—a nonexhaustive list [18].

Direct core complex protein interactions recruit multiple replication fork elongation factors (DnaB-Rep helicase interactions [37]), replication restart factors (DnaB-PriA [26]; Clamp loader-YoaA [38]), and DNA repair factors (DnaN-RecF [34]). Of primary relevance to the study of TRCs is the recruitment of the Rep helicase by DnaB [37,39]. DnaB, alone, is not capable of removing DNA-bound protein barriers [40]. This is likely due to the weak helicase activity of DnaB [41], with much of its processivity derived from interactions with other replication fork components [42]. Conversely, Rep can dislodge protein barriers *in vitro* [37,40,43,44] and Rep deletion *in vivo* is associated with decreased replication fork speed [45,46] and increased levels of endogenous DNA damage [47,48]. These replication defects in Δ*rep* are the result of TRCs, as removal of the Rep [49], or its *B. subtilis* counterpart—PcrA [50], RNAP interaction domain, recapitulates TRC phenotypes. Thus, while Δ*rep* mutants are viable and DNA replication can proceed in the absence of this helicase, Rep appears to be the first line of defense against transcription-induced replication stress *in vivo* [51] and many of the studies cited in this review use Δ*rep* strains as a sensitized system to study TRC interactions.

### The transcription complex

In bacteria, discussion of the transcription ‘half’ of TRCs cannot be simply limited to the RNA polymerase complex as RNAP directly interacts with post-transcriptional machinery such as the ribosome [52–54]. This RNAP-ribosome interaction is referred to as coupling, and this coupled complex has been referred to as the expressome [55], a term we will continue to employ here. It will be relevant to discuss the components and behaviors of the RNAP core enzyme, RNAP-associated factors, and the ribosome itself. Again, thorough reviews about the behaviors of each component are available [56–58]. Here, we will focus on RNAP elongation complex behaviors as most literature points to transcription elongation being a major contributor to replication fork disruption [59,60]. Conversely, non-specific RNAP-DNA complexes as well as RNAP-promoter complexes are easily dislodged by DNA helicases [61] and DNA-bound termination complexes are only weakly bound to their template [62], thus being unlikely to contribute to TRC phenotypes.

**Fig. 3.**
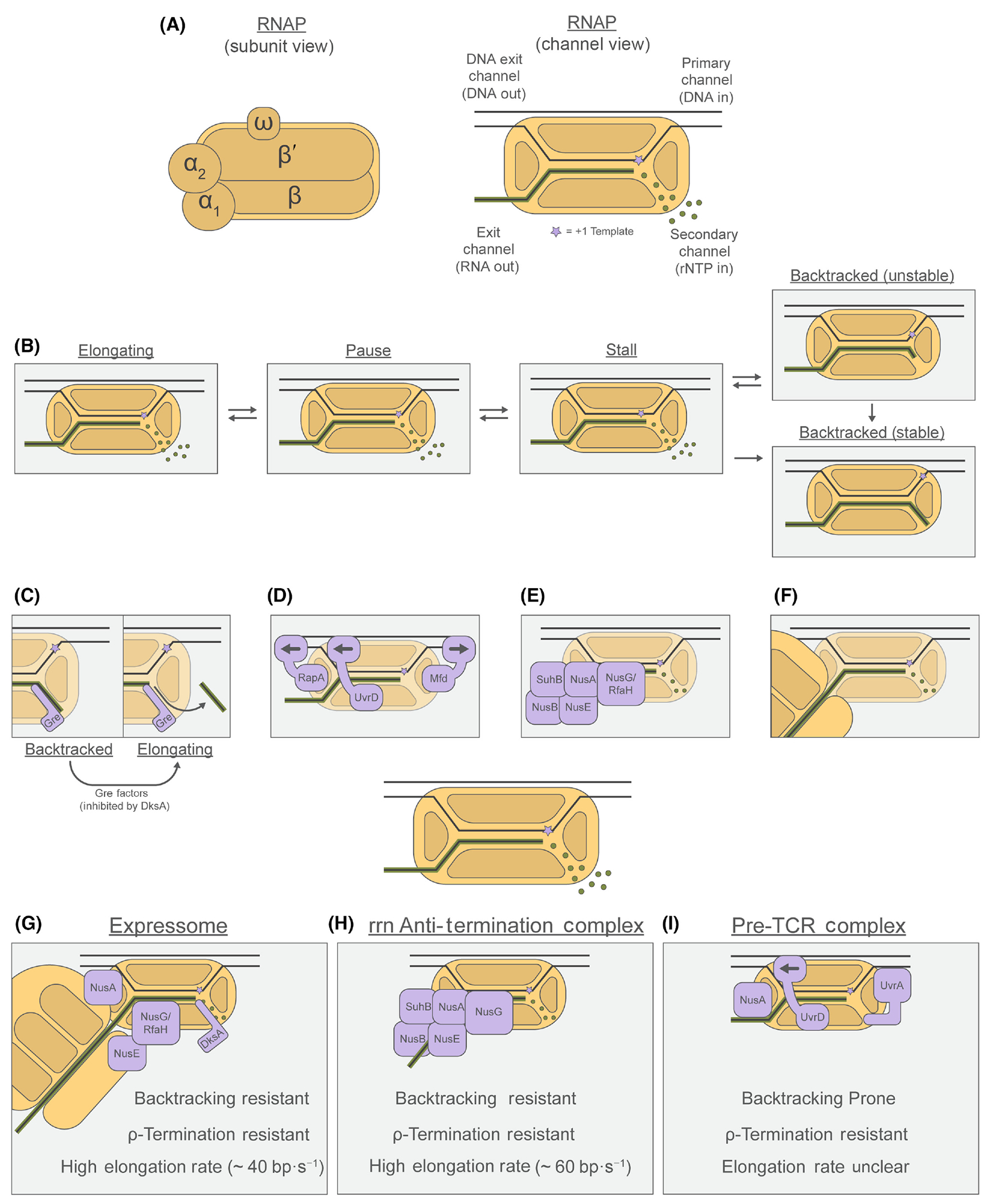
RNA polymerase is physically and functionally modular. (A) The RNAP core can be understood as a complex of physical components or a set of functional channels. In the physical component view, the enzyme is largely composed of its β and β′ subunits (which contain the enzyme’s ssDNA channel, catalytic site, and proofreading domain). The α subunit dimer and ω subunit are thought to play regulatory roles. In the functional channel view, RNAP can be simplified into an active center that contains the DNA–RNA hybrid and catalytic site as well as having DNA-in/out and RNA-in/out channels. These channels are frequently the interface for regulatory functions, thus, being important for understanding the *in vivo* behavior of the enzyme. (B) The RNAP enzyme engages in multiple functional conformations. These conformations appear to be in equilibrium with each other. Primary DNA sequence, regulatory proteins, and small molecule signals bias RNAP toward or away from specific conformations. (C–F) Multiple sets of regulatory factors act on RNAP to bias the enzyme’s activity and conformation preference. (C) Secondary channel factors can stimulate RNAP’s intrinsic RNA endonuclease activity to release the enzyme from backtracked states, biasing RNAP toward processive elongation. Secondary channel factors lacking co-endonuclease activity compete for this channel and bias RNAP toward a nonprocessive, backtracked state. (D) DNA helicases that bind RNAP translocate the enzyme forward or backward. These translocations force the enzyme into or out of processive states in a context-dependent manner. (E) A set of general transcription factors act in a context-specific manner to encourage/discourage factor-dependent transcript termination. (F) For coding transcripts, the ribosome translates nascent transcribed RNAs. Ribosome translocations create a physical and functional interaction between the ribosome and RNAP. (G–I) Sets of stable factor-RNAP interactions form functional complex subtypes with different composite behaviors. (G) The expressome is the primary RNAP subtype in protein-coding regions. Its primary feature is a tight ‘coupled’ interaction between the translating ribosome and RNAP. This interaction is stabilized by NusA–ribosome and NusG–ribosome interactions. The ribosome and secondary channel factor DksA cooperate to promote efficient RNAP elongation. (H) The rrn anti-termination complex is formed at ribosomal RNA loci. This complex is nucleated by primary sequence ‘boxes’. The assembled components coat the RNAP RNA exit channel, protecting the nascent RNA against premature termination and encouraging proper rRNA folding. (I) The pre-transcription-coupled repair complex is a genome-surveillance DNA repair complex that transcribes most of the genome, scanning for DNA lesions. Its components prevent factor-dependent transcript termination and sensitize the enzyme to pro-backtracking stimuli.

The RNAP core enzyme is a heteropentamer composed of two α (RpoA) and single β (RpoB), β′ (RpoC), and ω (RpoZ) subunits [63] (Fig. 3A, left). Functionally, the core structure can be understood as a catalytic center with four nucleic acid channels for DNA and RNA to enter and leave (Fig. 3A, right). This core complex acts in concert with any of a set of σ general transcription factors (σ^70^, σ^H^, σ^E^, σ^S^, σ^F^, σ^N^, σ^19^ in *E. coli*) to initiate RNA transcription [64]. Post-transcription initiation, the α_2_ββ′ω core sheds its σ factor [65] and assembles into one of a set of elongation complex subtypes, based on context-dependent protein recruitment described below. *In vitro*, this elongating α_2_ββ′ω core, alone, is proficient to synthesize some full-length RNA, as the core exhibits RNA polymerization and kinetic proofreading functions. Thus, the additional RNAP interactions we describe below are thought of as modifiers of transcriptional behaviors, rather than core to the process itself.

The RNAP core can enter multiple conformations that are relevant for understanding RNAP’s interaction with DNA replication forks. RNAP may processively elongate in an ‘elongation complex’, where it can exhibit short (< 1 s) pauses in a ‘paused complex’ [66,67], which can then extend into longer pauses in a ‘stalled complex’, where RNAP can then translocate backward into a ‘backtracked complex’ [68–70]. These backtracked complexes can then be further subdivided by their ability to escape from backtracking [71] ‘unstable backtracks’ *vs.* ‘stable backtracks’ [72], or the cause of backtracking ‘sequence-dependent backtracks’ *vs.* ‘misincorporation-dependent backtracks’ [73] (Fig. 3B). Each of these complexes (‘elongating’, ‘paused’, ‘stalled’, ‘backtracked’) appear to have different physical stabilities as judged by their temporal stability in bulk biochemical experiments [71,73,74] or, conversely, their behavior when force is applied with optical tweezers [68,75] or processive DNA helicases [61]. Extensive literature points to RNAP backtracking as a particularly stable conformation [76]—making it a barrier to incoming enzyme complexes, like a replication fork.

Most RNAP-interacting general elongation factors and helicases bias RNAP toward or away from specific conformations to effect behavioral changes in RNAP processivity or stability (Fig. 3C–F). The most obvious case of this modulation is with RNAP secondary channel interactors—which regulate RNAP backtracking [77] (Fig. 3C). During RNAP backtracking, the α_2_ββ′ω core translocates backward in reference to the underlying DNA template and nascent RNA 3′ end [69,70]. It appears that early (~ 1 bp) backtracks are reversible—if the enzyme translocates forward, the active site will realign with the 3′ end of the nascent RNA and elongation can resume. Longer backtracks lose the ability to resume elongation, suggesting entry into a mechanically distinct and stable state [71]. Without additional factors, RNAP will remain in this state permanently. However, a class of proteins called Gre factors can enter the RNAP secondary channel and cooperate with RNAP to cleave the dislodged RNA [74,77,78] (Fig. 3C). This leaves a new 3′ RNA end in the RNAP active site, allowing the enzyme to resume elongation. Thus, Gre secondary channel factors such as GreA act as anti-backtracking/pro-elongation factors [74]. Conversely, proteins or protein domains that insert into or block the RNAP secondary channel but lack co-catalytic RNA endonuclease activity, such as DksA, are expected to act as anti-anti-backtracking factors.

A second class of RNAP binding proteins consists of helicase domain-containing proteins that physically push RNAP, relative to the DNA template, which are termed translocases (Fig. 3D). These include UvrD, Mfd, and RapA. UvrD is a backward translocase which binds to the rear of the core enzyme, and forces the complex into a backtracked state [79], presumably from a previously paused conformation. RapA is a backward translocase that plays a role in post-termination RNAP recycling [80]. Mfd is a forward translocase that binds to the front of the core enzyme and forces the enzyme forward [81], which is an important step in RNAP restart after transcription-coupled nucleotide excision repair [82].

A third class of RNAP binding proteins are referred to as general transcription elongation factors such as NusA, NusB, NusG, RfaH, and SuhB (Fig. 3E). These proteins have pleiotropic functions and modulate the binding of RNAP to the ribosome (NusG-RpsJ bridge) [52–54], the binding of RNAP to repair factors (NusA-UvrD interaction) [82], regulate RNAP processivity (ribosomal RNA, rrn, anti-termination complex formation) [83–85] and pausing frequency (NusA/NusG antagonism) [86], and also facilitate transcription termination/anti-termination [87] (NusA/NusG interactions with transcription termination factor Rho) [88,89].

Finally, RNAP and the ribosome physically and functionally interact to form the expressome (Fig. 3F). Overall, the ribosome in *E. coli* functions as an anti-pausing, anti-backtracking factor for the following reasons. Decreasing the rate of ribosome elongation with ribosome inhibitors such as chloramphenicol results in a decreased average rate of RNAP elongation *in vivo* [90]. Nascent transcript sequencing reveals that RNAP frequently pauses early in coding gene bodies, after start codons—which suggests that elongating RNAP have been released from their pause by subsequent ribosome loading [91,92]. *In vitro* single-molecule experiments show that RNAP–ribosome complexes backtrack and correct transcription errors less frequently via both pause- or backtrack-dependent pathways [93]. Further *In vitro* studies have shown that ribosomes directly push RNAP out of unproductive states like pauses and backtracks [94], sometimes terminating the RNAP complexes altogether [95]. For the sake of understanding genomic stability, these studies collectively point to the ribosome as a pro-elongation factor for RNAP.

Binding to different combinations of these interacting factors creates functionally differentiated elongation complex subtypes (Fig. 3G–I). Current literature supports the existence of at least three major elongation complex subtypes *in vivo*: the expressome [55,96], the rrn anti-termination complex [97], and the pretranscription-coupled repair complex [82].

The expressome appears to be the standard subtype for the transcription of protein-coding genes (Fig. 3G). This complex forms from an RNAP α_2_ββ′ω core associating with NusA [65], NusG [65], Rho [65], DksA [98], and a ribosome [96]. NusG binds ribosomal protein RpsJ to form a bridge between the two complexes. DksA acts as a pro-elongation factor through an undefined pathway [98]. And NusA/NusG regulate transcription termination [99–101].

The rrn anti-termination complex subtype is thought to be specific to the transcription of rDNA loci and its formation is triggered by rDNA-specific primary sequence elements [102,103] (Fig. 3H). This complex is composed of an RNAP α_2_ββ′ω core associating with NusA, NusB, NusE, NusG, and SuhB [97]. As rDNA loci are not translated, there is no associated ribosome to inhibit premature Rho-dependent transcript termination, nor to inhibit RNAP from frequent pausing, stalling, and backtracking. Here, the rrn anti-termination complex appears to compensate for the lack of a ribosome by blocking premature termination [102,103] and encouraging high-speed transcription [104].

Finally, the transcription-coupled repair complex [82] subtype appears to function as a genome scanning tool in so-called pervasive transcription [105] (Fig. 3I). This complex is composed of an RNAP α_2_ββ′ω core associated with NusA, UvrA, and UvrD and is likely a small subpopulation of RNAP complexes [82]. This complex is not coupled to ribosomes, is Rho termination insensitive, and, due to a UvrA-secondary channel interaction as well as UvrD catalytic activity, is likely backtracking prone. This complex scans intra- and inter-genic regions via transcription, acting to detect lesion-based damage and initiate DNA repair [82,106].

The abundance of regulatory factors bound to RNAP, as well as the relative flexibility of transcriptional behavior (whereas a replisome is committed to a single function, elongation, during each replication cycle), makes RNAP a prime target for regulatory signal integration. The most well-known example is the interaction between RNAP and general starvation response ‘stringent response’ [107]. The initial stage of the stringent response is characterized by production of signaling nucleotide derivative called ppGpp [107,108]. ppGpp has two primary binding sites directly on the RNAP α_2_ββ′ω core, the first of which stabilizes an RNAP-UvrD probacktracking interaction [109] and the second of which stabilizes an RNAP-DksA interaction [110]. Beyond protein recruitment, ppGpp binding to RNAP binding site 1 increases the pause site sensitivity of RNAP [111]. Thus, regulatory signals can further modulate transcriptional behavior by modifying RNAP factor binding and behavior.

## Causes of deleterious transcription–replication collisions

### Genome-intrinsic stressors

**Fig. 4.**
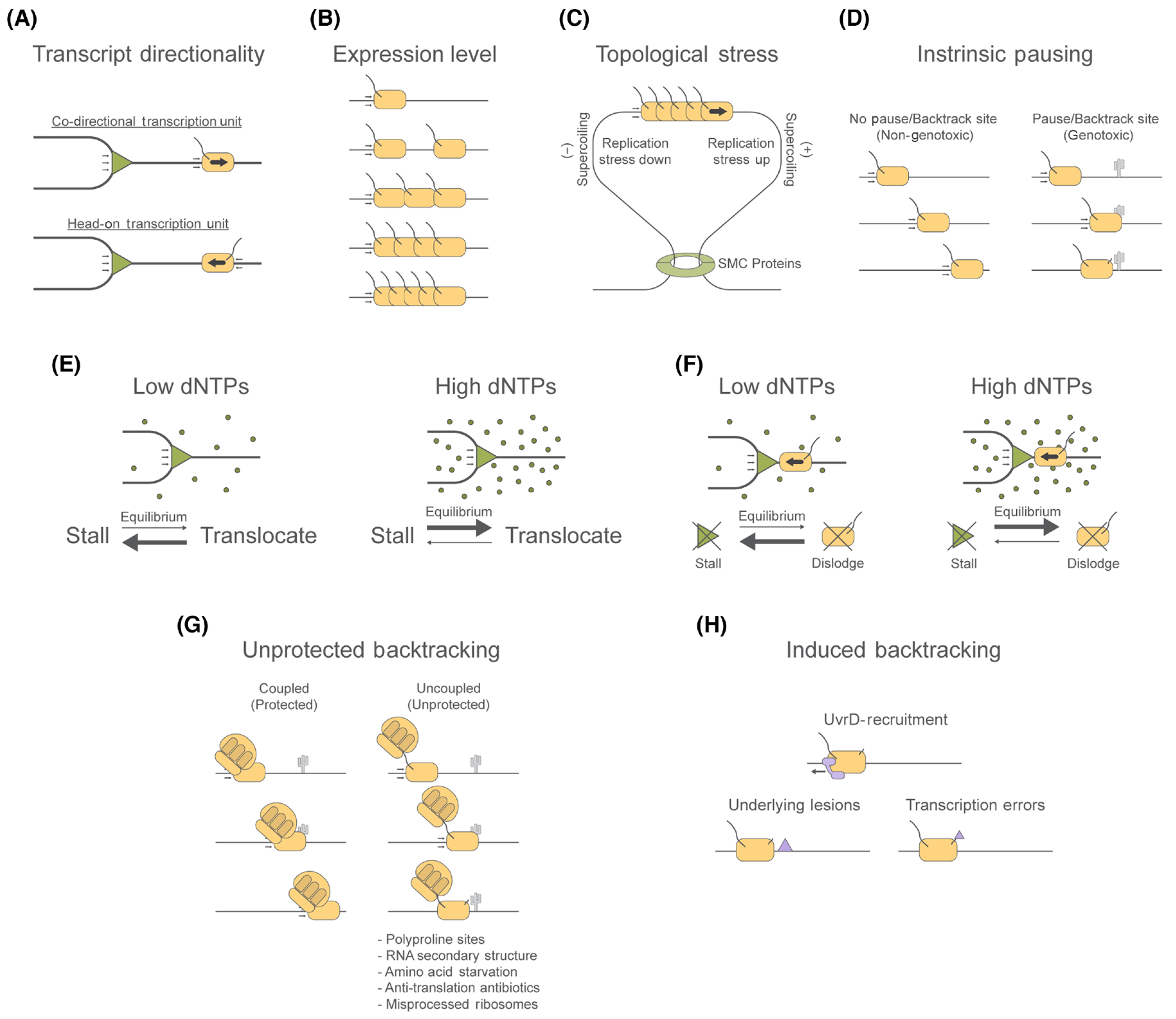
Causes of deleterious transcription–replication collisions. (A–D) Genome-intrinsic stressors. (A) RNAP–replisome interactions differ based on the directionality of RNAP, relative to the replisome (see Figs 6 and 7). (B) RNAP expression level alters the number of potential replisome barriers at a given locus. (C) As the genome forms constrained topological domains, transcription-derived supercoiling twin domains form potential static barriers to replication fork progression. (D) Intrinsic RNAP pause sites increase the frequency of RNAP pausing and backtracking, possibly enriching for transcription–replication collisions. (E, F) Replication fork dynamics are a function of dNTP concentrations (green dots), with low dNTP concentrations favoring fork stalling and high dNTP concentrations favoring fork forward translocation. By extension, dNTP levels are expected to alter the equilibria between collision-induced fork stalling and collision obstacle displacement. (G, H) Translation stressors converge in genotoxic RNAP backtracking by uncoupling the ribosome from RNAP, sensitizing RNAP to pause/backtrack signals. Other collision-inducing stressors converge on directly altering RNAP state to induce RNAP backtracking.

Evidently, most interactions between transcription and replication occur without causing DNA damage, replication fork collapse, or possibly even without causing replication fork stalling as removal of Rep unmasks a strong transcription-dependent DNA damage signal that is normally suppressed [37,44] and genome-wide analysis reveals replisome–transcription interactions in most transcription units in most cells [5]. With each bacterial genome containing thousands of transcription units, many of which are transcribed multiple times each replication cycle, most fork–RNAP interactions must be benign for cell survival or rapid replication to be possible. Experimental measurements of genomic damage estimate 10^−1^ to 10^−2^ damage events per cell division [109–111]. Estimates of fork collapse events are variable, but a number between 1 collapse [48,109] and 10 collapses [112] per replication cycle is reasonable. Observations of fork pausing events put this frequency at 10–100 pauses per cell division [113]. These low damage frequencies cannot be attributed to merely what fraction of transcription units are head-on with DNA replication as most bacteria contain ~ 10^3^ head- on genes per genome. As such, factors other than transcript orientation (Fig. 4A) or transcript expression level (Fig. 4B) must be considered.

One additional consideration is topological context (Fig. 4C). Although prokaryotic genomes are considerably smaller than most eukaryotes and are monochromosomal and circular for most, these genomes still form defined topological macrodomains (~ 800 kb) and domains (~ 15–30 kb) [112–114]. These smaller domains are expected to constrain the diffusion of chromosomal supercoiling [115,116]. Direct methods of detecting chromosomal supercoiling point to a nonuniform distribution of positive (overwound) and negative (underwound) supercoiling [117,118]. A large portion of supercoiling appears to be attributable to RNA transcription-induced ‘twin-domains’ [118,119]. The twin-domain model states that the RNAP expressome exhibits a large amount of inertia and thus will not rotate to alleviate transcription-induced topological stress [119]. This leads RNAP transcription to produce large amounts of positive supercoiling ahead of a given transcription unit and negative supercoiling behind a given transcription unit [120,121]. As upstream forces in DNA strands, such as that caused by positive supercoiling, can halt processive helicases [122], it is expected that the positive half of a strong twin domain could inhibit replication fork progression and predispose the complex to collapse [123]. This possibility is supported by the fact that slowing the resolution of replication-dependent positive supercoiling halts replication fork progression [124–126]. As such, DNA topology may explain a portion of TRCs.

An additional consideration is that underlying sequence context predisposes some transcription units to be particularly toxic to replication forks [127] (Fig. 4D). Known primary sequence determinants of RNAP pausing and backtracking have been reported [128,129]. As halted elongation complexes can stall leading strand DNA synthesis [130] and RNAP backtracked states are particularly stable—being reported to cause replication fork stress [60,131]—underlying pause/backtrack sites could predispose some loci to cause toxic replication fork collapse and subsequent DNA damage.

Together, the combination of gene body orientation, gene expression levels, chromosome topological constraints, and RNAP response to primary sequence context likely combine to account for baseline levels of TRCs. Further, these combined considerations may account for the heterogeneity of TRC-induced damage on a transcription-unit to transcription-unit basis. However, these considerations are under the assumption of relatively healthy cells living in stable, nutrient-abundant laboratory cultures. In nature, these cells are undergoing a myriad of stressors, which then modulate the propensity and distribution of TRCs.

### Replication stressors

Beyond signals caused by direct DNA damage, the DNA replication fork has a limited interface with changing stimuli. Across growth conditions and transcriptional programs, fork components and overall behavior appear to be unchanging [7]. However, the catalytic activity and forward translocation of DNA replication forks appears to be responsive to mass action relative to dNTP concentrations (Fig. 4E). That is, higher levels of dNTPs have been reported to result in faster fork translocation [132] which results in DNA damage [133] in yeast and decreased replication fidelity in yeast [132,133] and bacteria [134], whereas lower levels of dNTPs have been reported to result in slower fork translocation in yeast [133] and bacteria [47]. This fork responsiveness to varying dNTP concentrations is of physiological relevance as dNTP concentration has been reported to be modified by cellular stress responses [135–137].

This relationship between fidelity/mutation and dNTP levels may have created a stabilizing selection for cellular dNTP levels and, as such, dNTP pools are not in excess [138]. Thus, conditions of rapid DNA replication have been reported to cause DNA damage [139] and are rescued by increasing the pools of available dNTPs [140,141]. This suggests that existing dNTP pools are susceptible to transient replication-induced dNTP depletion. Further, genetic mutants that have decreased rates of replication initiation are resistant to partial dNTP depletion via treatment with the ribonucleotide reductase inhibitor hydroxyurea or the folate synthesis inhibiting antibiotic trimethoprim [142].

But why does modulation of fork speed/processivity impact TRCs? The crowded bacterial cytoplasm and nucleoid imposes high drag on large complexes [143], thus, momentum derived from speed is effectively irrelevant and processivity should be understood not as a physical phenomenon, but rather, as a chemical equilibrium (Fig. 4E). If so, a stalled fork can be thought of as being in equilibrium between dNTP addition and remaining stalled. An increase in dNTP levels will favor forward translocation, through blockage, rather than extended stalling and possible collapse (Fig. 4F). Although there is limited research on how external stressors affect DNA replication fork behavior, some studies suggest that conditions altering dNTP levels are likely to influence the abundance of transcription– replication conflicts.

### Transcriptional stressors

Most literature suggests that stressors altering transcriptional processes are the primary drivers of toxic TRCs. In this section, we will first discuss the responsiveness of ribosomal operons to environmental stimuli and the expected impact of this response on genome stability. Second, we will discuss the remaining coding genome with a focus on the impact of genome intrinsic (Fig. 4G) and extrinsic (Fig. 4H) sources of RNAP backtracking as the causes of genome instability.

Ribosomal operons are the most highly transcribed regions in any prokaryotic genome accounting for the activity of ~ 85% of active RNAP molecules and producing ~ 90% of cellular RNA [144]. These high levels of transcription result in the formation of RNAP arrays [104,145] that are expected to cooperate with each other to promote processive transcription [146]. Further, the formation of the rrn anti-termination complex with its increased transcription speed [104] and processivity [146] ensure that ribosomal transcripts are completed in a timely, uninterrupted manner [147]. Despite these protective measures, rDNA loci are associated with DNA damage [148] and correlate with markers of DNA damage, such as R-loops [149] and positive DNA supercoiling [118].

The genotoxicity associated with rDNA loci is expected to be the result of, and therefore function of, their high transcription rates. Under standard growth conditions, rDNA loci have low average RNAP occupancy and cells maintain the desired ribosome count through distributing transcription across multiple rDNA loci [150]. Decrease in the number of rDNA loci results in RNAP reallocation, with the remaining rDNAs experiencing elevated RNAP occupancy [104,149,150]. This elevated RNAP occupancy causes increased levels of R-loops, DNA damage, and drastically inhibits replication fork progression [148,149]. Thus, rDNA occupancy is correlated with replicationdependent DNA damage due to TRCs. Beyond experimental deletions, rDNA occupancy is known to be a function of cell growth rate [144], even when accounting for changes in rDNA copy number [8], as a function of chromosome replication levels. Cells with a doubling time of 100 min have an rRNA synthesis rate of 0.28 9 10^5^ nt·min^−1^ per rDNA locus, whereas cell with a doubling time of 20 min have an rRNA synthesis rate of 4.29 9 10^5^ nt·min^−1^ per rDNA locus – a 15-fold difference [144]. This means that rDNA-derived transcriptional stress is a function of cell growth rate, or nutrient availability [149]. This is functionally confirmed in experiments that reorient rDNA loci to be head-on with DNA replication [151,152]. In these experiments, cell viability and DNA damage are a function of media richness, with cells grown on minimal media experiencing significantly lower levels of DNA damage than their counterparts grown in rich media [151,152]. Occupancy of rDNA operons is further controlled by DksA-dependent stress response activation [153] providing a layer of rDNA regulation that is sensitive to cellular stress response activation [153] and relevant stress response mimics [154,155].

For coding transcripts, the relationship between cell growth rate and RNAP activity is considerably weaker. Cells with a doubling time of 100 min have an mRNA synthesis rate of 3.19 9 10^5^ nt·min^−1^ per genome equivalent, whereas cells with a doubling time of 20 min have an mRNA synthesis rate of 4.48 9 10^5^ nt·min^−1^ per genome equivalent—only a 1.4-fold difference [144]. Thus, the overall genotoxicity of protein-coding transcription is not expected to be directly related to cell growth or nutrient levels. However, existing literature points to clear examples of nutrient-dependent modulation of TRC levels and/or TRC toxicity [98,131,148,151,156,157]. The underlying mechanism appears to have less to do with overall transcription levels and more to do with the nutrient-induced changes in RNAP behavior or redistribution on the chromosome as described below.

As previously mentioned, RNAP largely takes on a complex subtype called the expressome when transcribing protein-coding regions [55,96]. The formation of this complex is associated with increased RNAP elongation rate [90], decreased RNAP pausing/backtracking behavior [91,93], and the termination of unproductive RNAP [94,95]. As such, coupling the functions of the ribosome and RNAP in the expressome protects RNAP from pausing/backtracking in response to underlying intrinsic pausing sequences [72,93,128] and difficult to transcribe regions [92]. As RNAP backtracking is toxic to DNA replication forks [60], this coupling is genoprotective and the absence of coupling [60] and RNAP termination [60,158] leads to replication-dependent DNA damage.

These genoprotective, pro-elongation functions are dependent on processive elongation of the ribosome, which, in turn, is dependent on an abundance of AA-tRNA molecules [159]. Increases in nutrient levels results in an abundance of AA-tRNA and processive translation; in contrast, lower nutrient levels result in a paucity of AA-tRNA and nonprocessive translation [160]. Low nutrient abundance can be mimicked in rich media by using amino acid hydroxamates, which inhibit AA-tRNA charging a specific amino acid (ex: serine hydroxamate blocks serine-tRNA^Ser^ charging) [161]. Treatment with serine hydroxamate causes chromosome replication to acutely arrest [131,162]. This replication arrest is associated with DNA damage and the severity of the arrest is dependent on the presence or absence of RNAP elongation factors, GreA, GreB, and DksA—independent of (p)ppGpp [131].

Chronic, physiological decreases in AA-tRNA abundance are detected by the ribosomal protein RelA [163], which, in turn, activates the stringent starvation response [164,165]. The stringent response signaling molecule ppGpp directly binds RNAP and modifies its behavior [109,110]. This ppGpp-RNAP interaction appears to relax transcription-induced DNA replication stress [131], indicating that the bacterial stringent response has evolved to soothe the genotoxic consequences of expressome uncoupling [156,157,166]. Additionally, under conditions of acute stress, ppGpp is known to arrest DNA replication initiation in *E. coli* [167] in a manner suggested to be dependent on *oriC* control proteins [162] and *oriC* supercoiling state [168]. ppGpp induction is also known to slow DNA replication elongation in *B. subtilis* [169], and to a lesser extent in *E. coli* [170], likely through a direct ppGpp–DNA primase interaction [171]. These interactions likely arrest DNA forks before fork–RNAP collisions can take place, providing a protective layer above direct ppGpp–RNAP interactions.

However, transient nutrient fluctuations, ribosometargeting antibiotics, translation-inhibitory RNA secondary structures, nascent protein poly-proline sites, and mis-processed ribosomes all are known to cause ribosome pausing [159,172]. These signals are perhaps not detected by the stringent response and should cause genotoxic expressome uncoupling. It appears likely that TRC toxicity is a function of these stressors that are not detected by cellular stress response networks, and as such are not compensated for. These undetected stressors are then expected to cause transient expressome uncoupling, subsequent paused/backtracked RNAP, and genotoxic replication stress (Fig. 4G).

The previously mentioned array of stressors all decreases the ability of the ribosome to protect RNAP from genome-intrinsic RNAP impediments, namely intrinsic pausing sites. However, an additional set of stressors/signals can induce backtracking events in RNAP in a manner independent, or largely independent, of primary sequence context (Fig. 4H). This includes RNA misincorporation-dependent backtracking [68,73,173], DNA lesion-dependent backtracking [174], and UvrD-dependent backtracking [79,109]. Current knowledge points to these stimuli as largely being stochastic, with RNA errors [175,176], DNA lesions [177,178], and UvrD activity (which is likely dependent on DNA lesions) happening at a basal rate that is a function of external stressors—such as oxidation-dependent nucleobase damage. Increases in these external stressors will then lead to a proportional increase in RNAP backtracking and subsequent replication stress.

### Stressor’s summary

Expected and confirmed replication stressors can be attributed to genome organization, replication fork catalytic activity, or RNAP behavior. The current body of literature suggests that underlying genome structure is important for determining the basal levels of TRCs, whereas variations in replication stress can largely be attributed to the behavior of RNAP and its subcomplexes. Analysis of RNAP behavior suggests that transcription-induced replication stress can be subdivided based on the transcription context, rDNA or coding loci. At rDNA loci, transcription levels/RNAP occupancy appear to be a major component of replication stress. Conversely, in coding loci, conditions of genotoxic stress seemingly converge on causing RNAP backtracking and with backtracked complex stability driving replications stress.

## Mechanisms of TRC-induced damage formation

Given that the predisposing conditions have been met—be it the wrong genomic context, a feeble replication fork, or a stubborn backtrack RNAP complex—it remains unclear what constitutes a ‘collision’, much less a ‘damage-inducing collision’. We will first address the topic of whether RNAP and the replisome interact directly or at a distance. Then, in each case, we will discuss models for ‘collision’-induced damage.

## Transcription–replication ‘collision’—a direct or indirect interaction?

**Fig. 5.**
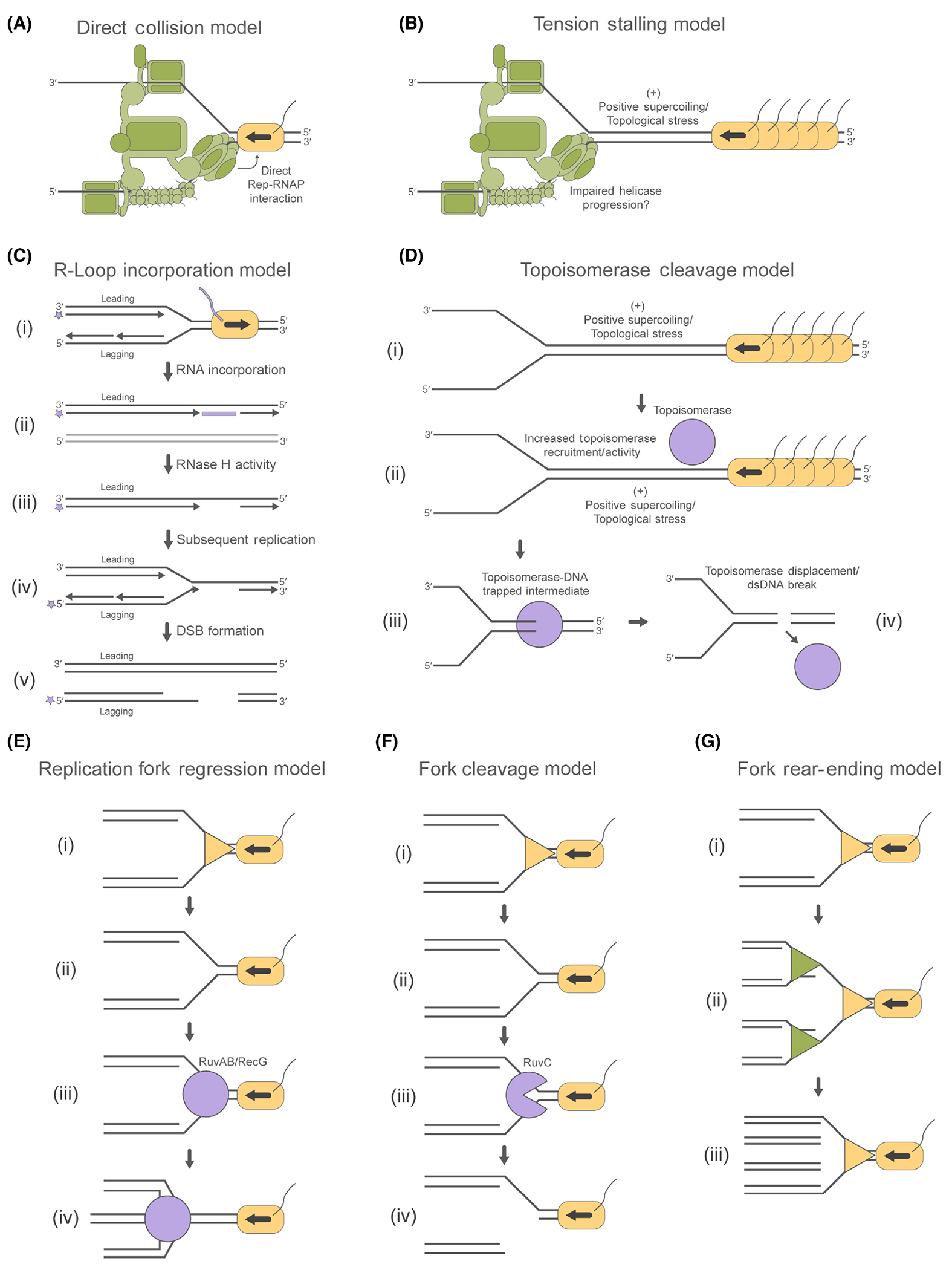
Models for collision-induced DNA damage. (A, B) Two overarching models for replisome-RNAP collisions exist that differ on whether the replisome and RNAP directly interact (A) or indirectly interact through supercoiling (B). (C) (i) The R-loop incorporation model depends on direct replisome-RNAP interaction. (ii) Upon collision, RNAP is dislodged and the nascent RNA is incorporated into dsDNA. (iii) RNase H removes the incorporated R-loop, leaving a single-strand gap in dsDNA. (iv) Subsequent DNA replication must occur prior to repair of the ssDNA gap. (v) If the replication fork passes the ssDNA gap before gap repair, the gap will be converted to a dsDNA break. (D) The topoisomerase cleavage model depends on indirect replisome-RNAP interactions via supercoiling. (i) Positive supercoiling builds up between the replisome and downstream RNAP. (ii) Supercoiling enhances topoisomerase recruitment. (iii) Topoisomerases form covalent dsDNA-protein attachments. (iv) Displacement of the topoisomerase-dsDNA intermediate leaves a dsDNA break. (E) The replication fork regression model is agnostic to direct/indirect interactions and also requires fork collapse. (i) The replisome stalls from a TRC. (ii) The replisome dissociates/collapses. (iii) Holliday junction migration helicases (RuvAB, RecG, etc.) associate with the junction. (iv) Forward junction migration is inhibited by the blockage and end of the nascent DNA strands. As such, the junction must be migrated backward. This allows nascent strand annealing to convert the three-way replication fork junction into a four-way junction with a dsDNA end. (F) The fork cleavage model is agnostic to direct/indirect interactions and requires fork collapse. (i) Replisome stalls from a TRC. (ii) The replisome dissociates/collapses. (iii) The exposed fork becomes substrate for endogenous DNA endonucleases. (iv) Cleavage of at least one strand results in a dsDNA end. (G) The fork rear-ending model is agnostic to direct/indirect interactions and only required fork stalling. (i) The replisome stalls from a TRC. (ii) Stalling of sufficient lengths of time allows subsequent rounds of DNA replication to ‘rear-end’ the stalled fork. (iii) Replication to the ends of the initial fork’s nascent strands converts them to two dsDNA ends.

The terminology ‘collision’ conjures an image of a molecular trainwreck, however, as discussed here in the Genome-intrinsic stressors section, when large complexes with helicase activity migrate across DNA, the combination of complex inertia and molecular crowding leads to DNA tension accumulation ahead of the helicase in the form of positive supercoiling. This supercoiling is generated by both replication fork progression [125] and transcription [118,120,121]. Positive supercoiling is known to inhibit replication fork progression [124,125]. Further, the accumulation of supercoiling coincides with the recruitment of replication fork restart proteins at head-on TRCs in *B. subtilis* [123,148]. This leads to the suggestion that supercoiling tension mediates fork arrest and collapse during ‘collisions’ (Fig. 5B).

In argument for a more direct interaction, the replication-associated helicase Rep has been shown to directly associate with the replication fork through a binding interaction between Rep and DnaB [37]. Removal of Rep appears to sensitize cells to TRCs [37,39,44], suggesting a role in TRC resolution. Further, it has been proposed that Rep dislodges barriers such as RNAP through direct interaction [49,50]. However, this model requires Rep to physically associate with the barrier in question. This is consistent with work that shows that DNA replication forks can stall directly in front of blockages (Fig. 5A), *in vivo*, but seemingly contradictory with TRC-at-a-distance models (Fig. 5B).

## How can these observations be reconciled?

### Synthesis model

Fork stalling is the result of supercoiling accumulation in the primary sequence dimension, but blockages are cleared due to the stalled fork and blockage being in close proximity in 3D space, permitting direct interactions between the blockage and fork-associated components. This could be enabled by supercoiled DNA plectonemes [179] which allow two anchors to be in close 3D proximity, while having a large length of coiled strand between them.

### Torque transmission model

Fork-associated helicases do not directly interact with blockages, and rather, dislodge barriers at a distance by increasing the torque exerted on supercoiled DNA. Transmission of torque across the supercoiling then dislodges the replication barrier.

### Topology context-dependence model

Supercoiling-mediated fork stalling is only relevant for the most highly expressed loci and is not relevant to the majority of the genome, where TRCs are mediated by a direct collision mechanism.

### Directionality context-dependence model

Supercoiling-mediated interactions at a distance are relevant for head-on TRCs, as codirectional TRCs do not appear to enhance topoisomerase recruitment, and helicase-mediated direct interactions are relevant for codirectional TRCs.

Current literature does not provide enough information to confidently reconcile these results. However, two types of damage have been observed that necessarily require direct replisome–RNAP interaction and indirect replisome–RNAP interaction, respectively.

## Direct interaction outcomes

**Fig. 6.**
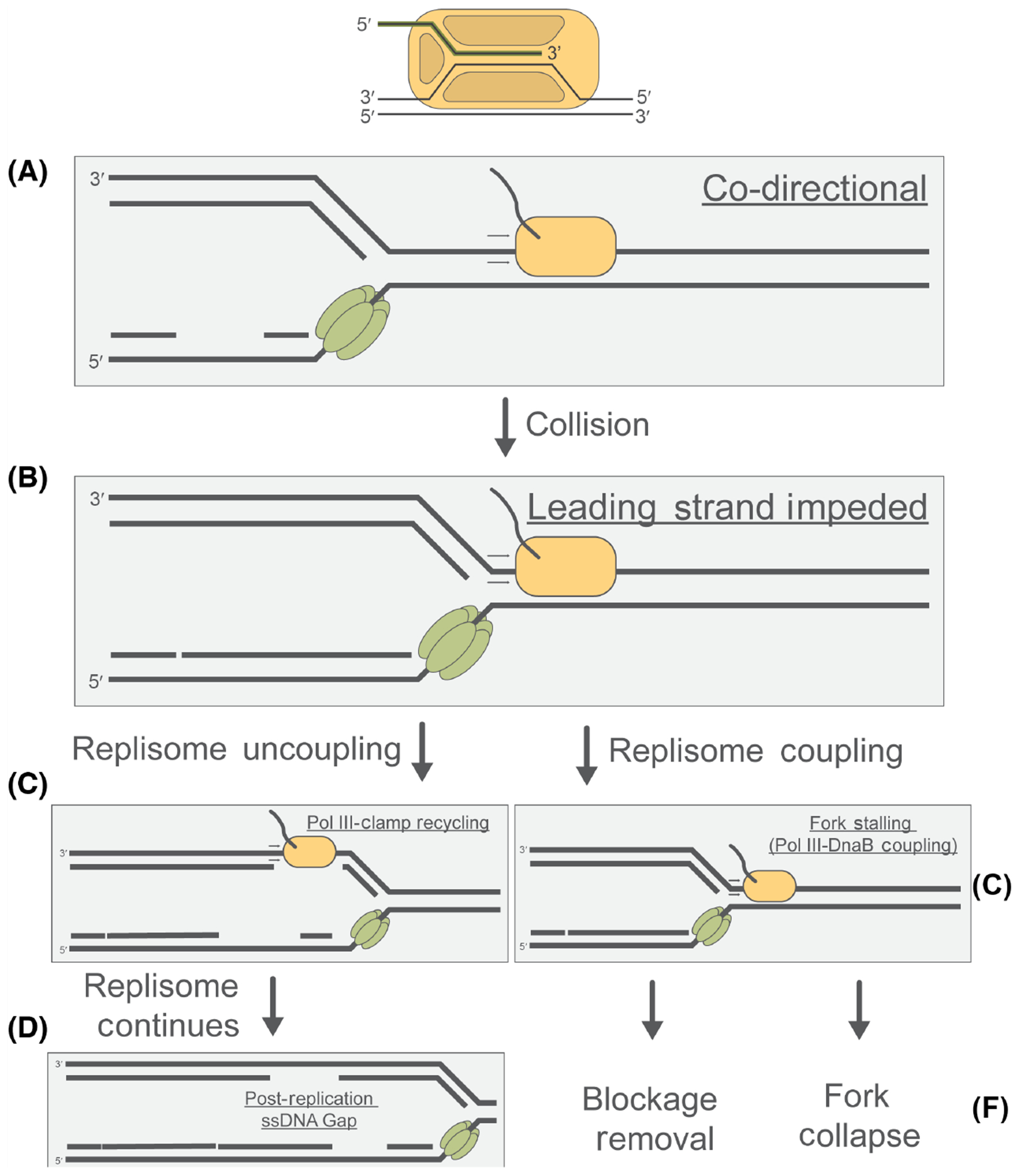
Molecular consequences of codirectional TRCs. (A) Codirectional TRCs place the replisome leading strand in the RNAP DNA channel, while leaving the DnaB-bound lagging strand exposed. (B) Contact of the replisome with RNAP stalls the leading strand DnaN-Pol III complex. (C) If DnaB and the leading strand DnaN-Pol III complex physically uncouple, the replication fork junction can pass RNAP and allow repriming of the leading strand DnaN-Pol III complex (see Fig. 5C: R-loop incorporation model). (D) Replisome progression is permitted and the location of the TRC is expected to have a ssDNA gap, which is subject to gap repair. (E) If DnaB-Pol III and DnaB remain physically coupled, the fork is expected to stall. (F) Fork stalling necessitates either removal of the RNAP blockage or collapse of the replication fork.

**Fig. 7.**
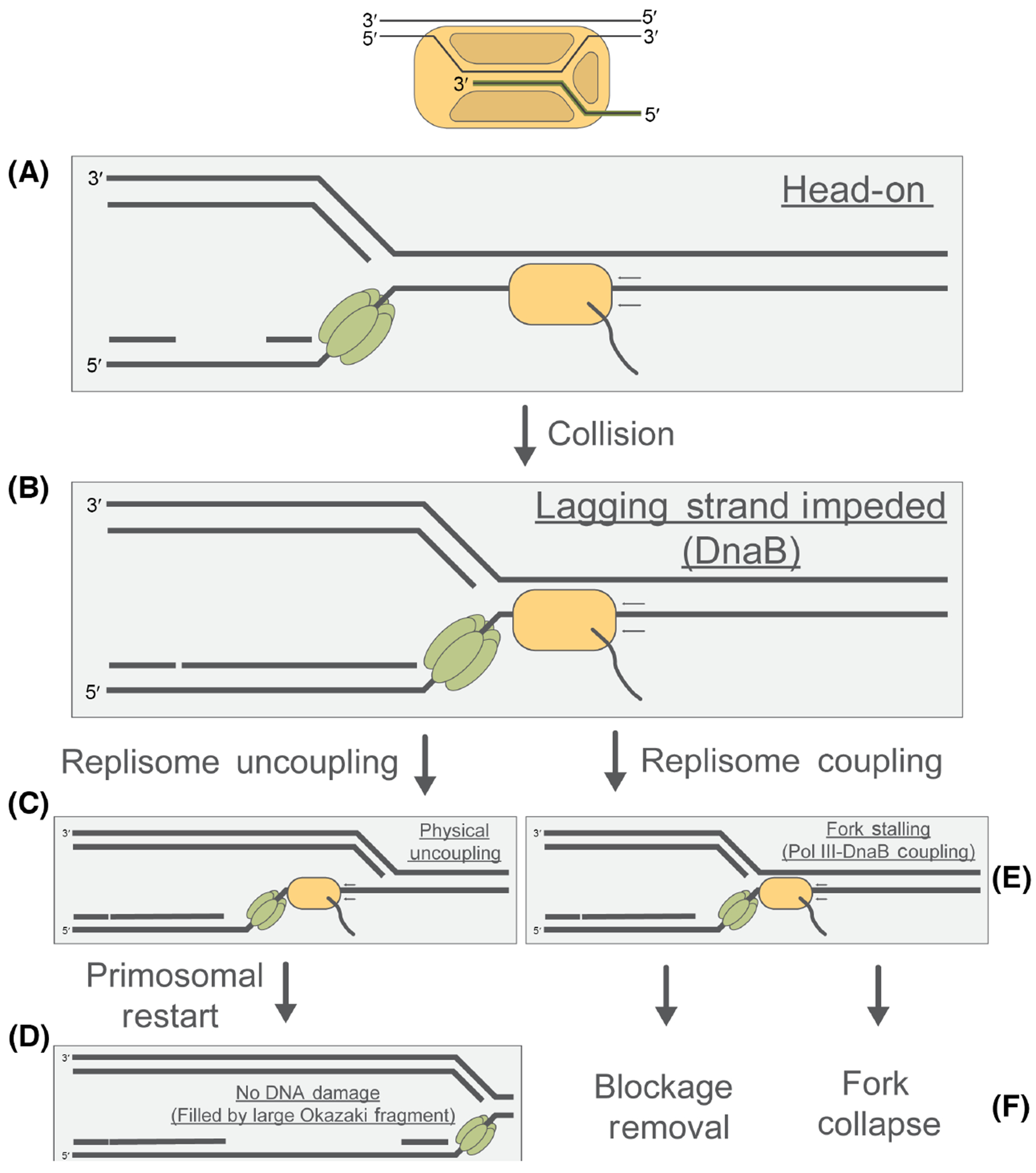
Molecular consequences of head-on TRCs. (A) Head-On TRCs place the replisome lagging strand in the RNAP DNA channel, while leaving the leading strand exposed. (B) Contact of the replisome with RNAP stalls the lagging strand DnaB helicase. (C) If DnaB and the leading strand DnaN-Pol III complex physically uncouple, the leading strand DnaN-Pol III could pass the blockage and act as a substrate for primosome-based replication restart. (D) Replication restart through this pathway is not expected to cause lasting DNA damage. (E) If DnaB-Pol III and DnaB remain physically coupled, the fork is expected to stall. (F) Fork stalling necessitates either removal of the RNAP blockage or collapse of the replication fork.

When direct interactions between RNAP and the replication fork are in question, it is important to understand that RNAP does not firmly bind to both DNA strands. Rather, the RNAP DNA channel only accommodates a single ssDNA strand corresponding to the template ssDNA [180]. This template strand is buried deep in the RNAP core. Here, it is tightly bound by a series of sequence-agnostic amino acid-DNA contacts [181], that when disrupted, decrease RNAP–ssDNA complex stability [182]. Conversely, the nontemplate strand is loosely situated within the clamp of the RNAP complex [180,181]. This loose positioning of the nontemplate DNA strand makes it an available substrate for enzymatic reactions. For example, in the case of T7 RNAP, which shares this clamp positioning of the nontemplate strand [183], convergently transcribing T7 RNAPs can pass by each other and continue transcription without dislodging each other [184]. Reports with *E. coli* RNAP differ, but collisions that do not result in stalling are not included in any prior analyses [185]. The directionality of replication and transcription synthesis then determines which strand is the source of conflict:

for codirectional TRCs, RNAP tightly holds onto the replication leading strand (Fig. 6A),

for head-on TRCs, RNAP tightly holds onto the replication lagging strand (Fig. 7A).

If direct contact is made between the replisome and RNAP, a codirectional TRC is a collision between RNAP and the Pol III complex (Fig. 6B), whereas a head-on TRC is a collision between RNAP and the DnaB replicative helicase (Fig. 7B). The differential outcome of these interactions is expected to be the product of a replisome phenomenon called replication fork coupling.

Replication fork coupling refers to the physical interaction between the three major fork components: the helicase, clamp loader, and polymerase. These three components are bridged through protein–protein interactions, primarily by the bridging protein tau [22–24,42]. This bridging affects the overall activity of the replication fork. In particular, this bridging alters the processivity of the replication fork [42]. The DnaB replicative helicase, alone, weakly [40] translocates at ~ 40 bp·^−1^ [41]. When coupled to the polymerase activity of Pol III, this speed increases to ~ 1000 bp·^−1^ [42]. This functional coupling explains the processive behavior of the DNA replication fork. As a result, blockage of either DNA strand could result in stalling of the entire replisome complex (Figs 6E and 7E) (see Stall-dependent (direct or indirect damage): the fork rearending model). Prolonged stalling then must be resolved by removal of the blockage or replication fork collapse (Figs 6F and 7F) (see Collapse/uncoupling-dependent (direct or indirect damage): the replication fork regression model and Collapse/uncoupling-dependent (direct or indirect damage): the fork cleavage model).

However, there have been reports that contradict the expected and observed coupling of the replisome. For example, the fork can steadily translocate forward even though leading strand Pol III frequently turns over [20,51]. This suggests that Pol III is not rigidly associated with DnaB. Additionally, single-molecule studies show that leading and lagging strand forward progression can transiently become independent [32], creating situations where—contrary to the name—the lagging strand moves ahead of the leading strand. Additionally, models for replication fork interactions with R-loops [130,186,187] (see the next section Direct interaction: the R-loop incorporation model) and DNA lesions [188,189] suggest that the lagging strand can translocate forward even when the leading strand is blocked. This forward translocation then allows repriming of the replication fork for leading strand blockages [130] (Fig. 6C,D). Also, uncoupling in the case of lagging strand blockage is expected to create the ideal substrate for the PriA-mediated replication restart pathway [26] (Fig. 7C,D).

Whether fork behavior can functionally be described as coupled or uncoupled is unclear. Realistically, the replisome probably acts in both ways, with coupling providing the mechanistic basis for processive translocation [42] but uncoupling providing flexibility [130,186–189] when dealing with fork component turnover and replication barriers. When interpreting *in vitro* experiments where replication forks are blocked on the leading strand (codirectional), it appears that replisomes blocked by leading strand impediments readily uncouple and bypass their blockage (on the scale of single minutes) [186]. Conversely, when dealing with lagging strand blockages (head-on), it appears that replisomes behave as if they were coupled and stall for long periods (on the scale of tens of minutes) [186,190,191]. This TRC context-dependence of repli- some coupling/uncoupling behavior may be the basis for the differential genotoxicity of codirectional and head-on TRCs. Further, when next discussing models for TRC-induced DNA damage, this TRC behavior determines which events must occur to form the substrate that leads to DNA damage.

## Direct interaction: the R-loop incorporation model

The R-loop incorporation model applies to replication forks that make direct contact with RNAP in a codirectional TRC orientation (Fig. 5Ci). As mentioned, this TRC results in stalling of the replisome leading strand Pol III enzyme. When this happens, Pol III leading strand synthesis is disrupted but eventually can continue in a manner dependent on displacement of blocking RNAP [130]. However, upon RNAP removal, an R-loop is still left intact, which alone can block replication fork progression [186,187]. But rather than removing the R-loop, Pol III can slide past the R-loop and use its 3′ hydroxyl to re-prime DNA synthesis [130] (Fig. 5Cii). This has been termed R-loop ‘takeover’. Alternatively, Pol III can dissociate and the replication fork primase can re-prime the leading strand ahead of the R-loop [186], providing an alternative priming point for Pol III.

The tendency of Pol III to take part in R-loop takeover or simply re-prime appears to be a function of the size of the R-loop in question and the complexity of the blockage [187]. If the R-loop is small and isolated, R-loop takeover appears to be efficient. If the R-loop is large, there are multiple R-loops, or there are multiple RNAP complexes present, repriming appears to be the dominant interaction [187]. Perhaps the tendency toward repriming can be explained by the mechanism of Clamp-Pol III release on the lagging strand, where loss of ssDNA ahead of the Clamp-Pol III complex results in destabilization and Pol III release [192].

It is interesting to note that repriming requires the DnaB helicase and associated DnaG primase to pass the obstruction and allow primase to re-prime the leading strand [186–188]. This suggests that even though Pol III supplies much of the translocating power for the replication fork [42], when Pol III is stuck on a leading strand obstacle, DnaB translocation can allow for obstacle bypass. And while it is clear that RNAP removal is necessary for R-loop takeover [130], it is relatively unclear that repriming requires RNAP removal.

However, regardless of R-loop takeover or repriming, any codirectional TRC that involves discontinuous leading strand synthesis will leave an ssDNA nick (Fig. 5Ciii). It is believed that this resulting nick can explain some of the genotoxicity of R-loops [60]. If the R-loop derived nick is unrepaired before subsequent rounds of DNA replication (Fig. 5Civ), this nick can be converted into a double-strand break (Fig. 5Cv), which may result in cell death.

Whether this strand break consequence of R-loop takeover or repriming occurs is unclear. Postreplication repair of the R-loop can occur through the standard Pol I pathway used to remove Okazaki fragment primers [193–196], Rnase HI-mediated R-loop degradation and subsequent Pol I gap filling [197], or the RecFOR ssDNA gap repair pathway [198]. The components of all three of these pathways are associated with the replication fork through direct interactions with Ssb [18] (see The Replisome section) (Fig. 2H). It is likely that these repair components make quick work of the resulting R-loop and nick—as is the case for Okazaki fragment integrated R-loops—unless specific inhibition of leading strand R-loop repair occurs.

## Indirect interaction: topoisomerase cleavage model

The DNA replisome creates large amounts of positive supercoiling ahead of the fork [125] (Fig. 5Di), which the replisome may deal with indirectly [199] or through the activity of DNA topoisomerases [200]. Topoisomerases, such as DNA gyrase, make DNA strand breaks, reorient the broken strands to alter the DNA supercoiling state, and reseal the strands [200]. Topoisomerases are actively recruited to the DNA replication fork [201], as well as regions of positive supercoiling caused by RNAP [123] (Fig. 5Dii). Presence of these topoisomerases assists in resolution of topological stress between the replisome and a blocking RNAP [123], which reduces the negative consequences of topology-mediated TRCs such as R-loop formation [123,202] and replication fork stalling/collapse (see the following three sections for mechanisms of stalling/collapse-induced DNA damage).

Though topoisomerases have a clear role in the maintenance of genome stability and avoiding replication fork barriers, the eukaryotic literature points to defects in topoisomerase function as a source of genomic damage [203]. There topoisomerases have been observed to form irreversible DNA-protein crosslinks or otherwise become interrupted during their catalytic cycle (Fig. 5Diii), with the end result being ssDNA and dsDNA strand breaks [203] (Fig. 5Div). Prokaryotic parallels can be seen in the study of antibiotic-induced topoisomerase-DNA intermediate stabilization. Quinolone class antibiotics such as nalidixic acid, norfloxacin, and ciprofloxacin arrest DNA topoisomerases such as DNA gyrase in the middle of their catalytic cycle, with a broken dsDNA intermediate [204]. This arrested intermediate is sufficient to cause replication fork arrest [205]. Arrested forks can then be cleaved by endonucleases [206] (see Collapse/uncoupling-dependent (direct or indirect damage): the fork cleavage model). Alternatively, helicases [207] can dislodge the stalled DNA gyrase molecule, converting the intermediate into a dsDNA break. These biochemical interactions suggest that topoisomerase recruitment ahead of replication forks, while necessary for DNA replication, are potential causes of genome instability if over-enriched or disrupted.

## Collapse/uncoupling-dependent (direct or indirect damage): the replication fork regression model

The replication fork regression model is based on the observation that the 3-way branch structure of a stalled DNA replication fork resembles the 4-way branched structure of a Holliday junction recombination intermediate [208]. When standard RecBCD homologous recombination pathways are disrupted, the formation of linear DNA or DNA damage in response to replication stress was dependent on the activity of the Holliday junction migration complex, RuvAB [209]. This could be explained by the RuvAB complex binding to stalled DNA replication forks and migrating them backward—reannealing the nascent DNA strands—to form a 4-way junction and free DNA end [210]. This is confirmed *in vitro*, where RuvAB catalyzes the regression of stalled DNA replication forks [211,212]. Later, RecG was also suggested to perform the same stalled-to-regressed fork function [156]. This RecG activity was later confirmed in bulk *in vitro* [211,212] and single-molecule studies [213,214], with the DNA contacts of a RecG-branched DNA structure [215] reinforcing a role for RecG in regressing stalled DNA replication forks.

The substrate specificities of RecG and RuvAB, for 3-way and 4-way junctions, respectively, suggest a model for replication fork regression, where RecG binds to an exposed DNA replication fork and initiates regression [211,212]. This initial regression creates the preferred substrate for RuvAB, permitting the more processive enzyme complex to further migrate the junction.

On a structural basis, binding of either RecG or RuvAB to a replication fork substrate requires direct access to the fork junction. This junction is normally stably occupied by the DnaB helicase (Fig. 5Ei). This competition for access to the fork junction appears to require replication fork collapse and DnaB dissociation (Fig. 5Eii) for RecG or RuvAB (Fig. 5Eiii) to initiate fork regression (Fig. 5Eiv). Though some *in vitro* regressed forks appear to maintain an intact lagging strand replication complex [211].

## Collapse/uncoupling-dependent (direct or indirect damage): the fork cleavage model

The fork cleavage model is based on the observation that certain DNA endonucleases can cleave fork-like structures [206,211]. This is because a collapsed (Fig. 5Fi) or collapsed and regressed replication fork takes on a Holliday junction-like structure, with 3 or 4 branches, respectively (Fig. 5Fii). This physical similarity to Holliday junctions lends the exposed fork to cleavage by Holliday junction resolvases, such as RuvC (Fig. 5Fiii). These resolvases normally function to cleave two ssDNA strands of a 4-way junction, and however, *in vitro* RuvC can cleave replication forks stalled by leading strand template lesions at a low frequency [211] (Fig. 5Fiv). This low frequency is enhanced by RecG or RuvAB, implying that RuvC activity at the fork is greatly enhanced following replication fork regression [211].

## Stall-dependent (direct or indirect damage): the fork rear-ending model

The fork rear-ending model is based on the observation that growing bacteria can undergo multiple cycles of DNA replication during the same cell division cycle, termed multi-forked DNA replication [8], and that the behavior of one set of DNA replication forks is seemingly independent of subsequent rounds of replication forks [7]. Thus, if one replication fork is stalled due to a TRC for long enough (Fig. 5Gi), subsequent forks will ‘rear-end’ the stalled fork [216] (Fig. 5Gii). The origin-proximal forks will generate free dsDNA ends, DNA damage, when they replicate to the end of the initial fork’s nascent DNA strands [210] (Fig. 5Giii). This model has been shown to occur in the context of long-lived replication blockages from ectopic replication termination protein, Tus, placements [216], rather than as a consequence of TRCs. Though, fork rearending could theoretically occur in any circumstance that results in long-lived fork stalling events. Thus, this model is suggested as a possible mechanism of DNA damage in cells undergoing over-initiation of DNA replication [47].

### TRC-induced damage summary

Current literature does not yet provide a unified mechanism for TRC-induced DNA damage or a clear understanding of replisome behavior during the TRC process. Further, of the possible damage mechanisms, the literature cannot clearly demonstrate which replication contexts lead to which damage mechanisms (i.e., which mechanisms are relevant to rDNA, as opposed to coding loci). Thus, we conclude that TRCs can occur through both distant topological interactions with or direct contacts with RNAP. For direct contacts, RNAP orientation can impact replication fork behavior, depending on models for physical coupling of the fork components. Finally, with various prerequisites for TRC readthrough, direct collision, fork stalling, or fork collapse, TRCs can cause double-stranded DNA damage through one of at least five distinct mechanisms. A proper assignment of which mechanisms predominate *in vivo*, as well as context-dependent assignment of DNA damage mechanisms is relegated to future work.

### Damage outcomes and repair

Transcription–replication collisions appear to be selected against, as inferred from nonuniform genome structuring (e.g., most genes are codirectional with DNA replication). One possibility is that TRCs cause a population-wide fitness defect by harming a subpopulation of cells that experience harmful TRCs. Another possibility is that TRCs and their potential to cause DNA damage that necessitates DNA repair, are mutagenic and can destabilize important genes. We will evaluate both possibilities and summarize current models for selection against harmful TRCs.

### TRC-induced cell death

**Fig. 8.**
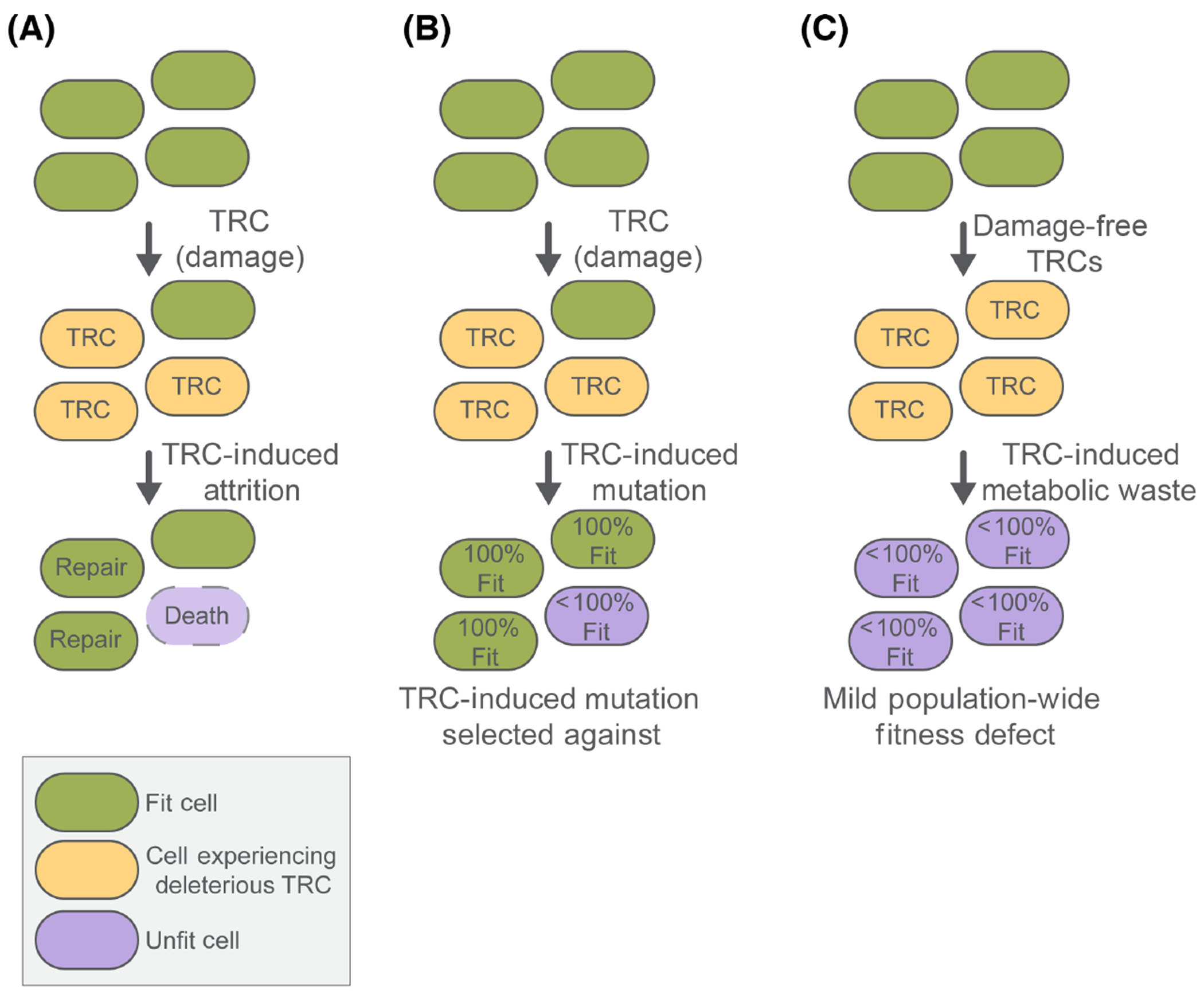
Physiological consequences of transcription–replication collisions. (A) One model for TRC counter-selection is based on the subset of TRCs that cause DNA damage. If unrepaired or improperly repaired, these TRCs could cause cell death. This resulting attrition places a selective pressure on a cell population to optimize their genome against TRCs. (B) Another model for TRC counter-selection is also based on toxic TRCs. This subset of TRCs is expected to be repaired by mutagenic mechanisms. Mutations in essential or otherwise important genes will cause fitness defects in a subset of the population that will be subject to counter-selection at the group level. (C) A final model for TRC counter-selection is based on the majority of TRCs which do not cause DNA damage, but rather cause premature termination of nascent RNA transcripts. These incomplete transcripts and all proteins derived from them will be destroyed. This presents the problem of TRC-induced metabolic waste that proportionally slows cell growth and depletes nutrients from the growth medium. This would be selected against.

A major model system for studying bacterial TRCs are strains where rDNA operons have been oriented such that they collide head-on with DNA replication forks—either through reorientation of the rDNA locus itself, to create *inv* strains [127,151], or through relocating the origin of replication, to create ectopic *oriC* strains [152]. Both *inv* and ectopic *oriC* strains experience cell death as a result of their rDNA TRCs [151,152]. This cell death is greatly increased during growth in rich media, leading to increased rDNA transcription and, therefore, more TRCs [151]. Thus, high levels of damage-inducing TRCs can result in cells that are at least inviable, if not outright dead. Such inviability, in theory, could provide a selective pressure that would result in a genome evolved to minimize TRCs, as we see in available natural genomes. However, no direct association has been made between genotypically ‘wild-type’, metabolically healthy cells and cell death from TRCs (Fig. 8A).

If the TRC phenotype that has been selected against is DNA damage, a proxy for TRC-induced damage is sensitivity to deletion of DNA repair components or induction of the SOS DNA damage response. Estimates of DNA damage based on repair mutant viability suggest that 25–50% of bacterial cells exhibit some form of DNA damage at any given time [217,218]. Estimates of DNA damage based on SOS induction dramatically reduce this number to ~ 1% [219]. Further, the majority (62%) of spontaneous SOS induction was RecB-pathway dependent [219]. We now know that the bulk of double-strand DNA damage, which is subject to processing by RecB, occurs due to fusing defects at the replication terminus [220,221]—which is likely not the direct result of TRCs. Of the cells with spontaneously activated DNA damage responses, ~ 2/3 were inviable [219]. If one assumes all viable cells are SOS^+^ from terminus defects, and all SOS^+^ inviable cells are the result of TRCs, this puts the upper limit of TRC-induced inviability at ~ 1in 300 cells. Realistically, DNA damage from oxidative damage, nucleotide excision repair, and other enzymatic pathways compose a portion of these SOS^+^ cells. Further, it appears that the likely cause of TRC stress in the first place—RNAP backtracking—assists in the repair of subsequent DNA damage [222], further mitigating the effects of TRC-derived DNA damage. Therefore, it is difficult to attribute the remarkable conservation of TRC-averse genome structure to TRC-induced cell death—as it is likely rare (Fig. 8A).

### TRC-induced mutagenesis

Events that alter the behavior of the DNA replisome, such as a TRCs, are expected to induce mutations through three broad pathways:

1. Direct modulation of DNA Pol III error rate at the site of collision.
2. Direct induction of mutations through mutagenic DNA break repair.
3. Indirect/diffuse mutagenesis through activation of stress-induced mutation pathways.

As elaborated in the previous section, TRC-induced damage, and therefore pathway 2: mutagenic break repair as well as pathway 3: stress-induced mutagenesis is expected to be exceedingly rare. This makes it unlikely that these pathways drive TRC-induced mutagenesis, leaving direct modulation of DNA Pol III error rate at the site of collision as the likely possibility.

This DNA Pol III centric mutation mechanism should produce mutations at the site of the TRC itself, allowing for the correlation of a genomic feature or gene with the mutations associated with its properties. Multiple genetic reporters of mutation show orientation-dependent changes in mutation rates relative to DNA replication [223–226]. These mutation patterns were correlated with bulk genome mutation datasets, suggesting that TRCs induce differential mutagenesis on the replication fork’s lagging strand [226]. The differential directionality of genes involved in bacterial pathogenesis, relative to the rest of the genome, led to suggestions that this differential evolvability was selected for to enable complex bacterial behaviors [227]. More recent studies have provided alternative explanations for the differential mutagenesis of codirectional *vs*. head-on oriented genes [228]. Further, analysis of spontaneous *E. coli* mutations, under neutral selection, shows no correlation of leading or lagging strand mutation rate, with gene orientation [177]. Most of these studies are restricted to the analysis of single base pair mutations and there is evidence for TRCs inducing duplications and deletions [224]—the broader effect of these complex mutations is unclear. The current state of the field suggests that TRC-induced mutation is not a major determinant of bacterial evolutionary patterns, with single base pair mutagenesis perhaps not present at all. Thus, broad and toxic TRC-induced mutagenesis is unlikely to be the selective pressure for anti-TRC evolutionary patterns (Fig. 8B).

### TRC-induced metabolic waste

As discussed in the Direct interaction outcomes section, multiple TRC outcomes require the displacement of the blocking RNAP complex. Early, direct observations of TRCs by electron microscopy clearly showed that RNAPs were displaced as a result of interactions with the replisome [229]. *In vitro* both head-on and codirectional TRCs required the removal of the offending RNAP for the replication fork to move forward [130,191]. And recently, aggregate single-cell analysis of division cycle-dependent transcription dynamics clearly shows that passage of a replication fork alters RNA transcript levels as the replisome passes the transcription unit in question [5].

These observations all point to the fact that each TRC, irrespective of the genomic damage outcome, results in a prematurely terminated transcript as well as a transient delay in the initiation of new rounds of transcription. For transcription units that rely on high levels of constant transcription, this disruption should have negligible effects on gene expression levels. However, genes that rely on relatively rare transcription events, if disrupted, would mimic transient knockdowns. Knockdowns of phenotypically important or essential genes will result in a transient fitness defect for the cell in question. This perspective of transcription–replication interactions predicts that the majority of TRCs will result in a mild, population-wide fitness defect. This fitness defect would be constant and provide a population-wide selective pressure to minimize the effects of TRCs on the genome. In lieu of a mechanism of TRC-induced cell attrition or toxic mutagenesis, this broad phenotypic effect is likely to drive the adaptive genome changes associated with TRCs (Fig. 8C).

### Eukaryotic contrast for TRC

The causes, nature, and resolution of TRCs in eukaryotes have several additional layers compared to those described in prokaryotes. Here, we highlight relevant and characteristic contrasts between eukaryotic and prokaryotic TRC induction, resolution, and evolution, to highlight their underlying differences.

## Contrast 1: Stochastic replication origin usage leads to stochastic TRC encounters

Unlike bacterial DNA replication, which initiates bidirectionally at a single and defined origin, *oriC*, many origins are required to replicate eukaryotic genomes in a timely manner, as they are larger and divided across multiple chromosomes. Mammalian cells can have around 30–50 000 origins [230]. Replication initiates from large megabase domains (~ 1 Mb in metazoans and ~ 100–200 bp in yeast) with clustered initiation zones within these domains [231]. Although multiple origins are contained within each zone, only a select few fire per cell division, conferring a significant level of stochasticity to origin firing. The excess number of origins compared to usage is thought to provide a failsafe against incomplete replication due to replication stress or to allow for coordination of cell-type specific gene expression programs to minimize TRCs. In systems with short cell cycles, such as in rapidly dividing embryos, more closely spaced origins are fired, to quickly complete replication [232]. Replication origins can be classified into three categories—constitutive, flexible, and dormant, the latter also provide redundancies to ensure the completeness of genome duplication [233]. Our current understanding is that origin choice is somewhat stochastic and cell-type dependent, allowing for adaptation to a wide array of conditions.

In most metazoans, origins are sequence-independent, although certain motifs have been associated with replication initiation. For example, the origin recognition complex (ORC) has preferences for supercoiled DNA and polyA-polyT tracts [234]. In mouse embryonic stem cells and fibroblasts, mouse teratocarcinoma cells, and Drosophila Kc cells, GpG-rich sequences coined OGRE (origin G-rich repeat elements) were found to be enriched at origins [235]. In contrast, yeast origins are AT-dense and in *Saccharomyces cerevisiae*, replication originates at 11-bp AT-rich elements called autonomous replication sequences (ARS).

This stochastic nature of origin location, usage, and firing pattern greatly complicates the analysis of genome evolution in response to TRCs. In prokaryotes, the simple location of the gene (left or right of *oriC* ) and coding strand (codirectional or head-on) determines the nature of that gene’s interaction with replication each cell cycle. This allows bacteria to consistently evolve against TRC-induced selective pressures. However, in eukaryotes, the same gene that was head-on with replication in the previous cell division or neighboring cell type can be codirectional with replication during this cell division or this cell type. Thus, a more detailed analysis is necessary to grasp the interaction between replication and transcription in eukaryotic models.

## Contrast 2: DNA replication and RNA transcription are temporally coordinated

Contrary to prokaryotes, eukaryotes break their cell cycle into defined periods, where the cell compartmentalizes cell growth and the various steps of DNA replication. A two-step process of replication initiation in eukaryotes helps prevent re-replication of DNA within the same replication cycle. This is done through a process called origin licensing, which occurs during mitosis and G1 phases. During origin licensing, ORC binds prereplication complex regions and then recruits CDC6/CDT1 and the replicative helicase MCM2-7 to form the pre-RC. The replicative helicase remains inactive until S phase origin firing, when the replicative polymerase is recruited. The factors and proteins involved in origin firing are well described in previous reviews [236–239]. We briefly focus below on the timing of origin firing and its role in TRC avoidance.

The restriction of DNA synthesis to S phase, allowing transcription to occur during the rest of the cell cycle is a potential mechanism to temporally separate transcription and replication, thus minimizing TRCs. However, in eukaryotes, there are more examples against this type of temporal separation than in support. This is because eukaryotic cells, due to their usage of large intronic regions for comparably small gene products, frequently must transcribe certain genes over the course of multiple cell cycle stages or entire replication cycles to obtain a single gene product. A classic example where temporal compartmentalization is impossible is the 2.2 Mb DMD locus, which takes over 16 h to transcribe, far longer than the 10-h cell cycle time reported for rapidly dividing human cells [240]. Additional large genes (defined as > 800 kb) take longer than a single-cell cycle to transcribe in B-lymphoblasts [241].

Early work suggested a possible compartmentalization of transcription and replication within S phase, itself. A general principle emerged that highly transcribed euchromatin is replicated in early S phase while poorly transcribed heterochromatin is replicated later in S phase [242]. However, broader study suggests that a conserved replication program is unlikely and is rather cell-type and environmental stimulus-dependent. One broad consensus is that replication origins are enriched near transcription start sites [243,244]. This positioning would naturally orient replication and transcription codirectionally, rather than head-on [245] and is perhaps a protective measure.

Development of genomic origin mapping techniques has refined our understanding of cell type-dependent origin firing. A recent single-cell, high-resolution map of replication speed in human RPE-1 cells used double pulse-label EdU incorporation to show that early replication does occur in highly transcribed regions [246]. However, during this time, fork progression is slower. As S phase progresses, replication speeds accelerate and the number of transcribed regions and overall gene expression levels decreases [246]. It has been largely presumed that the roughly equivalent speeds of eukaryotic replication forks and transcription complexes would avert codirectional TRCs. This study definitively shows that replication speeds can vary, even for a single cell over time and across the genome. Along with work in different organisms that estimate variability in Pol II elongation rates from cell-to-cell and within a single gene, the idea of a lack of codirectional TRCs is now clearly challenged [247,248].

## Contrast 3: Coding locus TRCs are transcription level-dependent

In bacteria, TRCs in rDNA loci are more strongly correlated to expression levels than in coding regions. This is potentially not the case in eukaryotes. In cancer cells, hyper-transcription from coding oncogenes leads to R-loop-dependent replication stress [249]. In human fibroblasts, overexpression of the HRAS^V12^ oncogene resulted in increased nascent RNA production and R-loop formation. This R-loop formation impedes replication fork progression and causes DNA damage. This series of events could be recreated by just the overexpression of TATA binding protein TBP, which mimics replication stress induced by oncogene hyper-transcription [250].

Hyper-transcription-induced R-loops and subsequent TRCs also appear to cause DNA damage through topoisomerase-dependent mechanisms. Eukaryotic topoisomerase I (Top1) relaxes supercoiling associated with replication and transcription. Human Top1-deficient cells have slow replication forks that are prone to pausing and accumulate DNA breaks in actively transcribed loci. These are rescued by RNase H, suggesting that cotranscriptional R-loops cause these replication impediments [251]. Camptothecin treatment, which binds the interface of Top1 and DNA to block DNA re-ligation, generates a reaction intermediate called Top1cc [252]. Within 2–5 min of camptothecin treatment, human cells accumulate Top1cc which potentiates TRCs. The mechanism of Top1cc-TRCs was recently shown through concerted genome-wide mapping of R-loops, DNA double-strand breaks, Pol II accumulation and replication origins in cancer cell lines [253]. Here, Duardo *et al*. demonstrate that stable Top1cc-dependent R-loops are formed in gene bodies and correlate with high transcription rates in early/mid replication zones of the S phase. Based on the requirement for the Pol II antibacktracking factor TFIIS, they propose that camptothecin-induced Top1ccs inhibit transcription elongation in coding regions, which causes backtracking. Backtracking provides an opportunity for R-loops to form downstream of the stalled Pol II, which arrests replication in the early-mid zones of the S phase. These arrests cause DSBs and micronuclei—hallmarks of genome instability. This solidifies a role for Top1 in preventing R-loop-dependent TRCs in highly transcribed genes [253].

## Contrast 4: An expanded toolkit to deal with R-loops

As discussed in the preceding sections, R-loop levels play a prominent role in eukaryotic TRCs. These R-loops form during transcription when the nascent RNA thread anneals to its own template DNA, which leaves the nontemplate DNA unpaired. This exposed nontemplate ssDNA is more susceptible to DNA damaging agents—one pathway of R-loops mediated genomic instability. In S/G2 cell cycle phases, these hybrids impede replication forks, causing a TRC. The resulting DNA damage presents a second pathway that R-loops threaten genome stability [254].

Programmed (or physiological) R-loops are generated in certain cells and have important functions. For example, large, > 1 kb stable R-loops occur in B lymphocytes. These are correlated with efficiency of class switch recombination at the IgH locus and hence diversification of antibody production during immune responses [255,256].

R-loops can be detected genome-wide by performing pull-downs with the S9.6 anti-10 nucleotide RNA–DNA monoclonal antibody and sequencing (DNA–RNA hybrid immunoprecipitation or DRIP-seq) [256]. By applying quantitative DRIP-seq to HeLa cells, where synthetic spike-ins were used to assess absolute R-loop abundance, it was estimated that human cells have approximately 300 R-loops at steady state. R-loop lifetime was assessed by transiently inhibiting transcription. For roughly 100 genes where half-lives could be fit to an exponential decay model, the mean half-life was 7–15 min. Combining the measurements of R-loop abundance and lifetime, it was determined that these cells resolve 18.9 R-loops per minute [257].

The presence of programmed R-loops alongside continual R-loop resolution suggests an R-loop homeostasis in eukaryotic cells. This homeostasis is maintained by (a) factors involved in R-loop formation and (b) factors involved in R-loop resolution.

### R-loop formation

Beneficial or physiological R-loops, as noted, have cell-type and context-dependent functions. One pathway of R-loop generation is mediated by the DDX1 helicase at the IgH immunoglobulin locus. Here DDX1 converts G-quadruplexes to hybrids that promote class switch recombination [258]. Another example of induced R-loop formation occurs at the telomeres. Here, the noncoding RNA TERRA (telomere repeat containing RNA) and RAD51-associated protein (RAD51AP1), support alternate telomere lengthening [259]. In this pathway, TERRA forms an R-loop with telomeric DNA which is stabilized by RAD51AP1. In cancer cells, RAD51AP1-TERRA association promotes repressive chromatin marks that may stall Pol II and prevent TRCs at telomeric DNA, permitting telomere lengthening and stability [260].

Several RNA processing and export factors may suppress R-loop formation by sequestering nascent RNA away from DNA. One example of this utilizes the RNA nuclear export machinery. One RNA export complex is TREX, which is composed of the THO complex, the DEXD-box RNA helicase UAP56/DDX39B, export adaptors (e.g., yeast Yra1) and several additional interactors [261]. Yeast mutants of THO complex component *hpr1* cause defects in transcription elongation due to rampant R-loop formation. These R-loop stalled RNAP complexes cause TRCs and subsequent hyper-recombination [262]. This has now also been shown in human cells lacking THO, where increased TRCs are suppressed by RNase H [263].

Other anti-R-loop factors act indirectly. For example, these factors may limit the formation of prolonged ssDNA regions, which are prone to hybridizing with RNA to form R-loops. One such factor is primase and DNA-directed polymerase (PrimPol). When replication forks are obstructed by polypurine repeats (10 copies of GAA) in avian DT40 cells, repriming by PrimPol is necessary for movement of forks through the offending region [264]. Without PrimPol, R-loops accumulate around repetitive DNA and genomic regions that are prone to forming secondary structures.

Conversely, RNA:DNA hybrids can be bound and stabilized by some factors including Yra1 in yeast, which when overexpressed can bind to R-loops and increase both head-on and codirectional TRCs [265]. In human cells, the m^6^A modification can positively and negatively impact hybrid levels through recruitment of other factors [266]. Reduced levels of m^6^A modification on noncoding RNAs can cause TRCs, exacerbated in cells where the H1 histone is depleted [267].

### R-loop resolution

Once formed, RNA:DNA hybrids can be removed by nucleolytic degradation, primarily catalyzed by RNase H enzymes. Eukaryotes frequently have two RNase H enzymes, RNase H1 and RNase H2, both of which degrade RNA in an RNA:DNA hybrid. RNase H2 is cell cycle-regulated, and functions during G2/M phases to degrade R-loops as well as incorporated rNMPs in the ribonucleotide excision repair pathway [268]. Conversely, RNase H1 is more relevant to TRC levels.

Several RNA helicases including Senataxin (Sen1/-SETX), which associate with actively replicating regions to clear R-loops as well as AQR, DDX19, yeast Pif1, DDX23, XXS1, Dbp2 (human DDX5) and Sgs1 (human BLM) also remove R-loops through their helicase activities [254]. RECQL5 is interesting among these as it forms a part of the Pol II elongation complex and reduces transcription processivity. By reducing Pol II backtracking and stalling, RECQL5 aids in TRC avoidance [269]. Additionally, RECQL5 associates with PCNA, the replicative sliding clamp. RECQL5 is considered a significant player in the regulation of TRCs. One mechanism based on its ability to bind to both replisome and transcription complexes proposes that the interaction between RECQL5 and PCNA at collisions recruits the TRIM28 SUMO E3 ligase leading to SUMOylation of PCNA. This leads to deposition of repressive histone marks in the vicinity, through recruitment of histone chaperones (CAF1 and FACT). Subsequent destabilization of Pol II and its removal from the DNA allows replication to continue [270]. Finally, RECQL5 also may act to minimize TRCs by preventing R-loop formation through its role in SUMOylation of topoisomerase I [271].

## Contrast 5: Genomic regions that are TRC-prone

While bacterial rDNAs are TRC hotspots, eukaryotes have additional known TRC hotspots. These are called fragile sites, where more DNA breaks and gaps occur following replication perturbation. Common fragile sites are distinguished from rare fragile sites, that are found in less than 5% of individuals [272]. Rare fragile sites are mostly related to sequence repeats such as di- and tri-nucleotide expansions.

Characteristics that distinguish common fragile sites include (a) late replicating, (b) origin poor, (c) tendency to be AT-rich and form secondary structures, (d) not identical between cell types, which is suspected to depend on cell-specific transcriptional programs (e.g., FRA16D is the most common CFS in lymphocytes, not found in fibroblasts), and (e) relatedly, they contain genes with long transcripts and their fragility is associated with transcription of those genes. These features underly the current hypothesis that the instability linked to fragile sites is a consequence of TRCs. Movement of an AT-rich region from the human gene *FRA16D*, that contains common fragile sites into the yeast genome caused replication stalling and DNA breaks at this region, supporting that the TRCs are responsible for propensity for damage [273].

A final class of sensitive genomic positions is called early replicating fragile sites (ERFs) that overlap with replicating origins. They are CpG rich and share a similarity to common fragile sites containing actively transcribed regions. These sites are more likely to experience DNA damage during replication stress [274].

## Conclusions

One confounding factor in drawing conclusions about TRCs is the aggregation of findings across different model systems. While it is overly pessimistic to claim that all conclusions must be limited to a single model, it is important to recognize that organisms have differentially evolved the conserved mechanisms of DNA replication and RNA transcription to suit their particular needs. These different model systems have undoubtedly evolved genetic background-dependent solutions to the same problems. This means that while new mechanistic insights can be considered across systems, the specific genetic background may render such mechanisms irrelevant. For example, the anti-pausing activity and lack of factor-dependent termination in *B. subtilis* RNAP may shift the focus from static modes of RNAP behavior, like backtracking in *E. coli*, to active modes of action, like supercoiling accumulation and R-loop formation.

Another confounding factor for drawing conclusions is the apparent context-specificity of replisome-RNAP interactions. From a simple, combinatorial standpoint, the replisome may collide with RNAP in 2 orientations, head-on and codirectional. Bacterial RNAP complexes have at least three known complex subtypes that can occupy at least 5 different functional conformations (as discussed in *The transcription complex*). Any of the 30 orientation-subtype-conformation combinations may be the primary driver of transcription–replication collision-induced stress. And as discussed in Transcription–replication collision—a direct or indirect interaction, different factors and collision mechanisms may apply to different replisome-RNAP interactions. Caution needs to be taken when applying results from one context to another. Studies in eukaryotes have identified various factors in different contexts and cell types that prevent and mediate TRCs. Future work is necessary to determine which factors are common and conserved versus unique adaptations to specific conditions across living organisms.

In lieu of these complications, what can we say about transcription–replication collisions with confidence?

1. Organisms are under selective pressure to avoid TRCs.
2. Codirectional TRCs are selected for, over head-on TRCs.
3. High transcription levels enrich for TRCs in rDNA loci in prokaryotes.
4. Transcription complex conformation influences TRC toxicity in coding loci.
5. TRC resolution could involve the removal of the transcription complex.
6. Cellular metabolic state influences TRC levels.

In the past 40 years, the study of transcription–replication collisions has transitioned from postulating about their existence to studying their physiological relevance. We have built an understanding of who the molecular players are, the many possible ways that these players can interact, and how the cell can deal with the consequences of these interactions. However, there is still a significant gap in our understanding, between what can happen and what does happen. Further studies will allow us to discern the context and genome-specific realities of transcription–replication collisions and map these realities onto the biology of natural cells.

## Figures and Tables

**Fig. 1.**
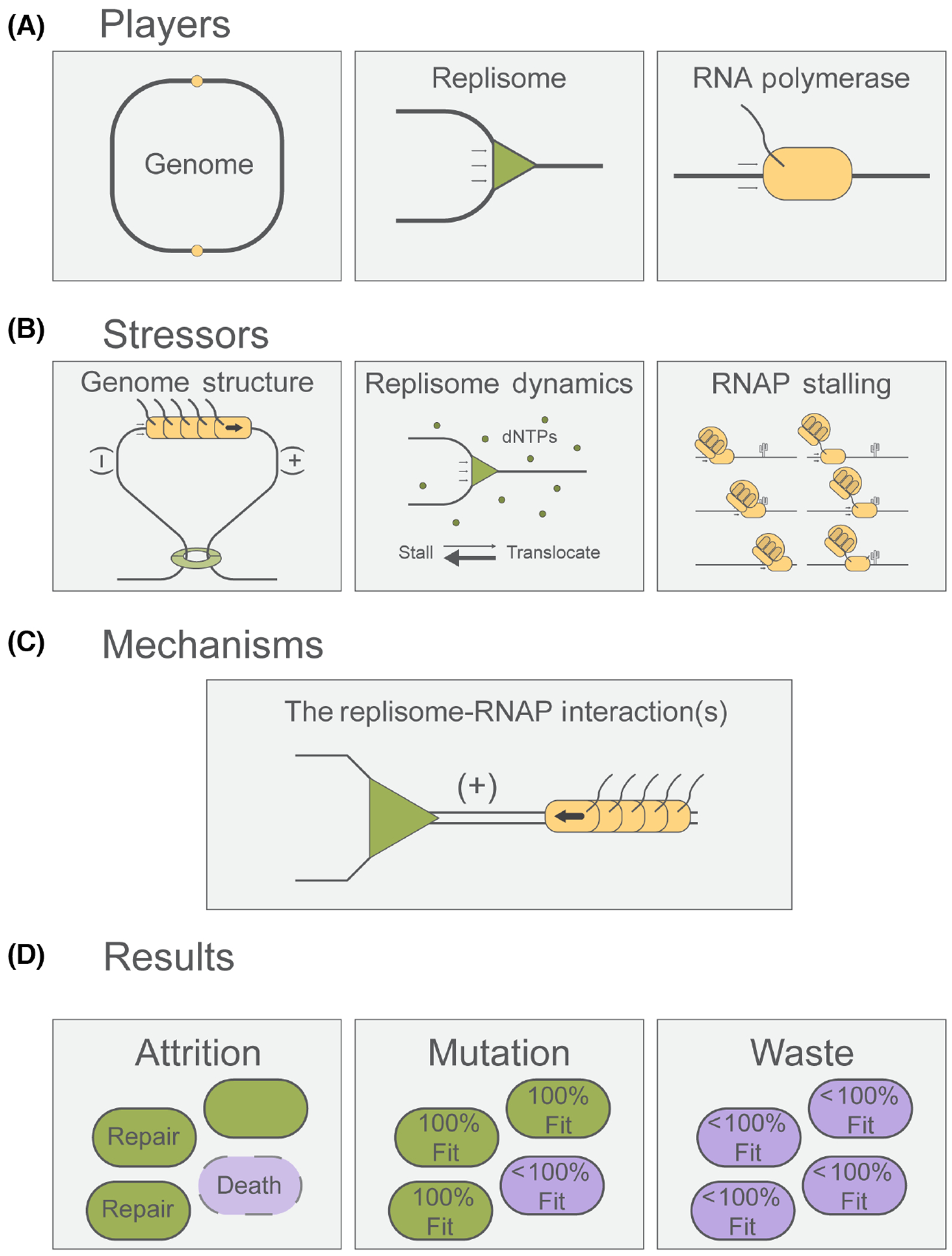
Review overview. (A) TRCs can be best understood as a three-way interaction between underlying genome structure, the replisome complex, and the RNA polymerase complex. (B) Intrinsic and external stressors exacerbate the inherent contributions of the genome, replisome, and RNAP to toxic TRCs. (C) The behaviors of the genome, replisome, and RNAP result in multiple different possible TRC outcomes. (D) TRCs impose a fitness cost on the cell through either causing DNA damage, DNA mutagenesis, or metabolic waste.

## Abbreviations

HR: homologous recombination
RNAP: RNA polymerase
TRC: transcription–replication collision
